# Supplementary material for: HiC-bench: comprehensive and reproducible Hi-C data analysis designed for parameter exploration and benchmarking
Source: BMC Genomics. 2017 Jan 5;18:22. doi: 10.1186/s12864-016-3387-6 (PMC5217551; doi:10.1186/s12864-016-3387-6)
Supplement: Additional file 1: — HiC-Bench Manual. (PDF 3853 kb) [file 12864_2016_3387_MOESM1_ESM.pdf]

HiC-Bench Manual

Stephen M. Kelly [1,2], Charalampos Lazaris [3,4,5], Aristotelis Tsirigos [1,2,3,4,5]

[1] Applied Bioinformatics Center, Office of Collaborative Science, NYU School of Medicine, NY 10016, USA

[2] Genome Technology Center, Office of Collaborative Science, NYU School of Medicine, NY 10016, USA

[3] Department of Pathology, NYU School of Medicine, New York, New York 10016, USA

[4] NYU Cancer Institute and Helen L. and Martin S. Kimmel Center for Stem Cell Biology, NYU School of Medicine, New York, New York 10016, USA

[5] Center for Health Informatics & Bioinformatics, NYU School of Medicine, NY 10016, USA

March 22, 2016

Contents

1 Introduction **2**
- 1.1 Installation . . . 2
- 1.2 Compile Binaries . . . 2
- 1.3 Setting up a new analysis . . . 2
- 1.4 Setting input files . . . 3
- 1.5 Create project sample sheet . . . 3
- 1.6 Running the Pipeline . . . 3
- 1.7 Dependencies . . . 5

2 Default Pipeline Components **6**
- 2.1 Parent Directory Overview . . . 6
- 2.2 Code Directories . . . 7
- 2.3 Data Directory . . . 7
- 2.4 Inputs Directory . . . 7
- 2.5 Pipeline Directory . . . 8

3 Custom Pipeline Steps **12**
- 3.1 Overview . . . 12
- 3.2 How To Add Steps . . . 12

4 HiC-Seq Pipeline **19**
- 4.1 Pipeline Steps . . . 19
- 4.2 Alignment . . . 22
- 4.3 Filter . . . 23
- 4.4 Filter Stats . . . 24
- 4.5 Tracks . . . 27
- 4.6 Matrix Filtered . . . 29
- 4.7 Matrix Prep . . . 31
- 4.8 Matrix IC . . . 32
- 4.9 Matrix HiCNorm . . . 33
- 4.10 Matrix Stats . . . 34
- 4.11 Compare Matrices . . . 36
- 4.12 Compare Matrices Stats . . . 38
- 4.13 Boundary Scores . . . 41
- 4.14 Boundary Scores PCA . . . 42
- 4.15 Domains . . . 44
- 4.16 Compare Boundaries . . . 45
- 4.17 Compare Boundaries Stats . . . 46
- 4.18 HiC Plotter . . . 49
- 4.19 Interactions . . . 51
- 4.20 Annotations . . . 53
- 4.21 Annotations Stats . . . 54

5 Appendix **56**
5.1 Error Logs . . . 56
5.2 gtools-hic . . . 56
5.3 pipeline-master-explorer . . . 59
5.4 System and Session Information . . . 60

List of Figures

1 Example sample sheet . . . 4
2 Overview of pipeline step execution for default analysis steps. See Section 4.1.2. . . . 21
3 Filter Stats counts sample output . . . 25
4 Filter Stats percentage sample output . . . 26
5 WashU tracks loaded in browser. . . . 28
6 Matrix Stats sample output . . . 35
7 Compare Matrices Stats Spearman sample correlograms. See Section 4.12. . . . 39
8 Compare Matrices Stats Pearson sample correlograms. See Section 4.12. . . . 40
9 Boundary Scores PCA sample output. See Section 4.14. . . . 43
10 Example raw comparisons. See Section 4.17. . . . 47
11 Example correlograms. See Section 4.17. . . . 48
12 HiCPlotter sample output . . . 50
13 Interactions sample output . . . 52
14 Annotation Stats enrichment sample output. See Section 4.21. . . . 55

1 Introduction

1.1 Installation

The analysis pipeline can be installed by cloning its git repository from GitHub, located here: `https://github.com/NYU-BFX/hic-bench`. In the Terminal (OS X, Linux), run a command such as the following:

```
git clone https://github.com/NYU-BFX/hic-bench.git
```

Once a clone of the pipeline repository has been made, it will be used as a blank template to start future analysis; analysis is not performed directly in the pipeline repository.

1.2 Compile Binaries

Source code for needed binaries has been included in the repository, and must be compiled. Navigate to the `code/src` directory from within the Terminal, and run the command `make` to automatically run the compilation scripts needed. The program `bedGraphToBigWig` is also required, and available as a binary file from UCSC at their page here: `http://hgdownload.cse.ucsc.edu/admin/exe/`. To directly install a version compatible with the Linux operating system, navigate to the `code/bin` directory and run the command `wgethttp://hgdownload.cse.ucsc.edu/admin/exe/linux.x86_64/bedGraphToBigWig`.

1.3 Setting up a new analysis

Assuming your pipeline repository clone exists at `~/hic-bench`, use the following terminal command to create a new analysis:

```
~/hic-bench/code/code.main/pipeline-new-analysis hicseq-standard <project_name>
```

This will create a new directory at the given location, and copy into it all the basic files and sub-directories needed for analysis from the pipeline repository.

1.4 Setting input files

Manual setup for the pipeline input files requires the creation of the directories `<project_name>\pipeline\input\fastq` or `<project_name>\pipeline\input\bam`, corresponding to the type of input files to be used. Sub-directories within these should be created with the name of each sample to be included in the analysis. A naming scheme similar to the following is suggested:

```
<Cell_line>-<treatment>-<SampleID>
```

Importantly, the '-' should be used as a delimiter, since this is recognized by the sample sheet creation script. Within each sub-directory, place all fastq / fastq.gz or bam files for the sample. Symlinks can be used if the files are not contained in the same location as the project analysis directory, and are preferable in order to save storage space. Since this part of the pipeline setup is custom for each analysis, it must be completed manually. A script used to automatically create the correct directories and symlinks might look like this:

```
#!/bin/bash
Fastq_dir="/data/sequence/results/smithlab/2016-01-28/fastq"
Inputs_dir="/home/$(whoami)/projects/SmithLab_HiC_2016-02-09/inputs/fastq"

# make inputs dir
mkdir -p "$Inputs_dir"

for i in $Fastq_dir/*.fastq.gz; do
  echo "$i"
  TMP_NAME=$(echo "$(basename "$i")" |sed -nr 's/^([[:digit:]])[^[:alnum:]]([[:alpha:]]+)[^[:alnum:]].*$/THP1
     -\2-\1/p' )
  echo "$TMP_NAME"
  mkdir -p "$Inputs_dir/$TMP_NAME"
  ln -s "$i" "$Inputs_dir/$TMP_NAME"
done
```

1.5 Create project sample sheet

A sample sheet must be created for the analysis project. After the inputs directory has been set up, the follow command can be used to automatically create a sample sheet template:

```
inputs$ ./code/create-sample-sheet.tcsh <genome> <fragment-size>
```

Where genome is hg19, hg38, etc.. The fragment-size entry is optional and should be a numeric argument such as 300, representing the library size of the sequencing sample. After creation of the sample sheet (`sample-sheet.tsv`), a manual review process is required to match the correct control or input samples with experimental samples, verify proper grouping names, files, and other entries. If not entered prior, fragment-size should be filled in for each sample. This process can be completed within Microsoft Excel, but saving the file in Excel should be avoided due to the introduction of invisible formatting errors by Microsoft Office products. It is advisable to instead copy the finalized sheet from Excel and paste directly into a terminal text editor such as vi or nano for saving under the file name `sample-sheet.tsv`.

1.6 Running the Pipeline

After navigating to the parent directory of the analysis project, run the pipeline with:

```
./code.main/pipeline-execute PROJECT-NAME E-MAIL
```

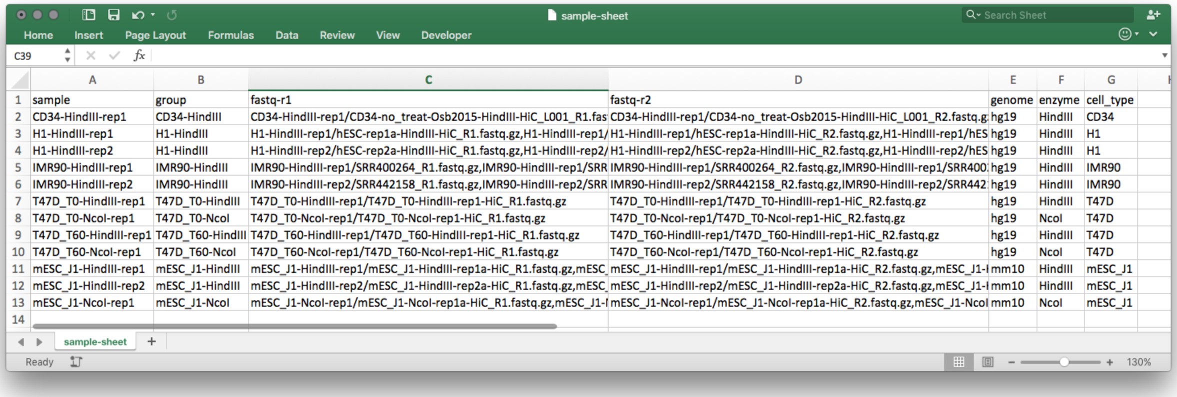

	sample	group	fastq-r1	fastq-r2	genome	enzyme	cell_type
2	CD34-HindIII-rep1	CD34-HindIII	CD34-HindIII-rep1/CD34-no_treat-Osb2015-HindIII-HiC_L001_R1.fas	CD34-HindIII-rep1/CD34-no_treat-Osb2015-HindIII-HiC_L001_R2.fastq.g	hg19	HindIII	CD34
3	H1-HindIII-rep1	H1-HindIII	H1-HindIII-rep1/hESC-rep1a-HindIII-HiC_R1.fastq.gz,H1-HindIII-rep1/	H1-HindIII-rep1/hESC-rep1a-HindIII-HiC_R2.fastq.gz,H1-HindIII-rep1/hES	hg19	HindIII	H1
4	H1-HindIII-rep2	H1-HindIII	H1-HindIII-rep2/hESC-rep2a-HindIII-HiC_R1.fastq.gz,H1-HindIII-rep2/	H1-HindIII-rep2/hESC-rep2a-HindIII-HiC_R2.fastq.gz,H1-HindIII-rep2/hES	hg19	HindIII	H1
5	IMR90-HindIII-rep1	IMR90-HindIII	IMR90-HindIII-rep1/SRR400264_R1.fastq.gz,IMR90-HindIII-rep1/SRR	IMR90-HindIII-rep1/SRR400264_R2.fastq.gz,IMR90-HindIII-rep1/SRR400	hg19	HindIII	IMR90
6	IMR90-HindIII-rep2	IMR90-HindIII	IMR90-HindIII-rep2/SRR442158_R1.fastq.gz,IMR90-HindIII-rep2/SRR	IMR90-HindIII-rep2/SRR442158_R2.fastq.gz,IMR90-HindIII-rep2/SRR442	hg19	HindIII	IMR90
7	T47D_T0-HindIII-rep1	T47D_T0-HindIII	T47D_T0-HindIII-rep1/T47D_T0-HindIII-rep1-HiC_R1.fastq.gz	T47D_T0-HindIII-rep1/T47D_T0-HindIII-rep1-HiC_R2.fastq.gz	hg19	HindIII	T47D
8	T47D_T0-NcoI-rep1	T47D_T0-NcoI	T47D_T0-NcoI-rep1/T47D_T0-NcoI-rep1-HiC_R1.fastq.gz	T47D_T0-NcoI-rep1/T47D_T0-NcoI-rep1-HiC_R2.fastq.gz	hg19	NcoI	T47D
9	T47D_T60-HindIII-rep1	T47D_T60-HindIII	T47D_T60-HindIII-rep1/T47D_T60-HindIII-rep1-HiC_R1.fastq.gz	T47D_T60-HindIII-rep1/T47D_T60-HindIII-rep1-HiC_R2.fastq.gz	hg19	HindIII	T47D
10	T47D_T60-NcoI-rep1	T47D_T60-NcoI	T47D_T60-NcoI-rep1/T47D_T60-NcoI-rep1-HiC_R1.fastq.gz	T47D_T60-NcoI-rep1/T47D_T60-NcoI-rep1-HiC_R2.fastq.gz	hg19	NcoI	T47D
11	mESC_J1-HindIII-rep1	mESC_J1-HindIII	mESC_J1-HindIII-rep1/mESC_J1-HindIII-rep1a-HiC_R1.fastq.gz,mESC_	mESC_J1-HindIII-rep1/mESC_J1-HindIII-rep1a-HiC_R2.fastq.gz,mESC_J1-	mm10	HindIII	mESC_J1
12	mESC_J1-HindIII-rep2	mESC_J1-HindIII	mESC_J1-HindIII-rep2/mESC_J1-HindIII-rep2a-HiC_R1.fastq.gz,mESC_	mESC_J1-HindIII-rep2/mESC_J1-HindIII-rep2a-HiC_R2.fastq.gz,mESC_J1-	mm10	HindIII	mESC_J1
13	mESC_J1-NcoI-rep1	mESC_J1-NcoI	mESC_J1-NcoI-rep1/mESC_J1-NcoI-rep1a-HiC_R1.fastq.gz,mESC_J1-	mESC_J1-NcoI-rep1/mESC_J1-NcoI-rep1a-HiC_R2.fastq.gz,mESC_J1-NcoI	mm10	NcoI	mESC_J1

Figure 1: Example sample sheet

1.7 Dependencies

This pipeline was developed for use in a High Performance Computing environment, running CentOS 6. Additionally, tcsh and bash shells are required, along with R version 3.2.0. The following includes software used in the HiC-Seq pipeline:

```
OGS/Grid Engine 2011.11
Linux 2.6.32-573.3.1.el6.x86_64 #1 SMP Thu Aug 13 22:55:16 UTC 2015 x86_64 GNU/Linux
tcsh 6.17.00 (Astron) 2009-07-10 (x86_64-unknown-linux)
GNU bash, version 4.1.2(1)-release (x86_64-redhat-linux-gnu)
armatus/2014-05-19
bedtools/2.22.0
bowtie2/2.2.6
caltads/0.1.0
ghmm/0.9
java/1.7
matlab/R2013a
picard-tools
python/2.7.3
r/3.2.0
r/3.2.3
samtools/1.2.1
```

The following R packages are used in the pipeline:

```
plyr 1.8.1
VennDiagram 1.6.16
flsa 1.05
genlasso 1.3
ggplot2 1.0.1
optparse 1.3.0
pastecs 1.3-18
plotrix 3.5-11
reshape2 1.4.1
zoo 1.7-12
preprocessCore 1.24.0
MASS 7.3-35
gplots 2.17.0
reshape 0.8.5
corrplot 0.73
RColorBrewer 1.1-2
lattice 0.20-33
grid
stringr 1.0.0
```

For software information specific to the creation of this document, see Section 5.4

2 Default Pipeline Components

2.1 Parent Directory Overview

A default pipeline will have the following basic structure within its parent directory:

```
hicseq.analysis-for-hicbench$
lrwxrwxrwx  1 at570  14 Feb 14 19:28 __01a-align -> pipeline/align
lrwxrwxrwx  1 at570  15 Feb 14 19:28 __02a-filter -> pipeline/filter
lrwxrwxrwx  1 at570  21 Feb 14 19:28 __02b-filter-stats -> pipeline/filter-stats
lrwxrwxrwx  1 at570  15 Feb 14 19:28 __03a-tracks -> pipeline/tracks
lrwxrwxrwx  1 at570  24 Feb 14 19:28 __04a-matrix-filtered -> pipeline/matrix-filtered
lrwxrwxrwx  1 at570  20 Feb 14 19:28 __05a-matrix-prep -> pipeline/matrix-prep
lrwxrwxrwx  1 at570  18 Feb 14 19:28 __06a-matrix-ic -> pipeline/matrix-ic
lrwxrwxrwx  1 at570  23 Feb 14 19:28 __07a-matrix-hicnorm -> pipeline/matrix-hicnorm
lrwxrwxrwx  1 at570  21 Feb 14 19:28 __08a-matrix-stats -> pipeline/matrix-stats
lrwxrwxrwx  1 at570  25 Feb 14 19:28 __09a-compare-matrices -> pipeline/compare-matrices
lrwxrwxrwx  1 at570  31 Feb 14 19:28 __09b-compare-matrices-stats -> pipeline/compare-matrices-stats
lrwxrwxrwx  1 at570  24 Feb 14 19:28 __10a-boundary-scores -> pipeline/boundary-scores
lrwxrwxrwx  1 at570  28 Feb 14 19:28 __10b-boundary-scores-pca -> pipeline/boundary-scores-pca
lrwxrwxrwx  1 at570  16 Feb 14 19:28 __11a-domains -> pipeline/domains
lrwxrwxrwx  1 at570  27 Feb 14 19:28 __12a-compare-boundaries -> pipeline/compare-boundaries
lrwxrwxrwx  1 at570  33 Feb 14 19:28 __12b-compare-boundaries-stats -> pipeline/compare-boundaries-stats
lrwxrwxrwx  1 at570  19 Feb 14 19:28 __13a-hicplotter -> pipeline/hicplotter
lrwxrwxrwx  1 at570  21 Feb 14 19:28 __14a-interactions -> pipeline/interactions
lrwxrwxrwx  1 at570  20 Feb 14 19:28 __15a-annotations -> pipeline/annotations
lrwxrwxrwx  1 at570  26 Feb 14 19:28 __15b-annotations-stats -> pipeline/annotations-stats
lrwxrwxrwx  1 at570  30 Feb 18 11:16 code -> code.repo/code.hicseq-standard
lrwxrwxrwx  1 at570  14 Nov 12 11:55 code.main -> code/code.main
drwxr-xr-x 10 at570 238 Feb 15 19:53 code.repo
lrwxrwxrwx  1 at570  36 Mar 10 16:30 data -> /ifs/home/at570/pipeline-master/data
drwxr-xr-x  5 at570 230 Jan  5 09:24 inputs
drwxr-xr-x 25 at570 834 Feb 14 19:28 pipeline
-rwxr-xr-x  1 at570 981 Jan  5 19:40 run
-rwxr-xr-x  1 at570 554 Dec 18 17:02 run.dry
```

The following components can be seen here:

- `__01a-align ... __15b-annotations-stats`: Symlinks to each step in the pipeline, in alphanumeric order of execution.
- `code`: Symlink to the directory containing scripts and code specific to the current analysis type e.g. ChIP-Seq.
- `code.main`: Symlink to the directory containing scripts and code used for all pipelines.
- `code.repo`: Directory containing all code for the project, copied from the main pipeline repository.
- `data`: Symlink to a directory containing reference genome data; set this in your original repository clone.
- `inputs`: Directory containing information on the files used as inputs.
- `pipeline`: Directory containing the files needed for each step in the pipeline.
- `project_notes`: A bare directory in which you can place miscellaneous notes and documents concerning the analysis.
- `run`: File containing code for running the pipeline.
- `run.dry`: File containing code for testing the pipeline without execution of pipeline steps.

2.2 Code Directories

The code needed for the execution of the analysis pipeline is divided among several sub-directories, based on usage. Within an analysis pipeline, the directory `code.repo` contains all of these sub-directories.

```
hicseq.analysis-for-hicbench/code.repo$
drwxr-xr-x  2 at570   434 Dec 30 11:47 bin
drwxr-xr-x  3 at570  1.7K Feb 15 19:53 code.chipseq-standard
drwxr-xr-x  3 at570  2.4K Feb 15 13:28 code.hicseq-standard
drwxr-xr-x  2 at570  2.1K Feb 15 19:53 code.main
```

- `bin`: A directory containing symlinks to binary files for programs used by the pipeline.
- `code.chipseq-standard, code.hicseq-standard`: Directories containing scripts specific to the execution of each step in the the given type of pipeline analysis.
- `code.main`: A directory containing code and scripts used for all analysis pipelines.

2.3 Data Directory

The reference genome information needed for analysis is contained in the `data` directory. This can be contained in an external location and symlinked to the project directory if it has not already been set in the cloned HiC-bench repository template. Our example `data` contains only the subdirectory `genomes`, which is configured as such:

```
data/genomes/hg19$
-rw-r--r--  1 at570 at570   36M Nov 23 12:43 HindIII.fragments.bed
-rw-r--r--  1 at570 at570  298M Mar  7 15:58 MboI.fragments.bed
-rw-r--r--  1 at570 at570   32M Nov 23 12:43 NcoI.fragments.bed
lrwxrwxrwx  1 at570 at570    20 Nov 23 12:41 bowtie2.index -> genome/bowtie2.index
-rw-r--r--  1 at570 at570  1.5K Nov 23 13:43 centrotelo.bed
drwxr-xr-x  2 at570 at570  1.2K Mar  7 16:00 features-hicnorm
-rw-r--r--  1 at570 at570  2.2M Dec 30 22:58 gene-name.bed
-rw-r--r--  1 at570 at570  2.2M Nov 30 15:43 gene.bed
lrwxrwxrwx  1 at570 at570    33 Nov 23 12:34 genome -> /ifs/home/at570/Data/Genomes/hg19
-rw-r--r--  1 at570 at570   564 Nov 23 13:43 genome.bed

data/genomes/mm10$
-rw-r--r--  1 at570 at570   36M Nov 23 12:47 HindIII.fragments.bed
-rw-r--r--  1 at570 at570   37M Nov 23 12:47 NcoI.fragments.bed
lrwxrwxrwx  1 at570 at570    20 Nov 23 12:47 bowtie2.index -> genome/bowtie2.index
-rw-r--r--  1 at570 at570  1.3K Nov 23 13:48 centrotelo.bed
drwxr-xr-x  2 at570 at570  1.2K Mar  7 16:00 features-hicnorm
-rw-r--r--  1 at570 at570  1.4M Dec 30 22:58 gene-name.bed
-rw-r--r--  1 at570 at570  1.4M Nov 30 15:43 gene.bed
lrwxrwxrwx  1 at570 at570    33 Nov 23 12:47 genome -> /ifs/home/at570/Data/Genomes/mm10
-rw-r--r--  1 at570 at570   495 Nov 23 13:48 genome.bed
```

Also included are indexes for `bowtie2`, which can be obtained from `bowtie-bio.sourceforge.net/bowtie2/manual.shtml` or `http://support.illumina.com/sequencing/sequencing_software/igenome.html`.

2.4 Inputs Directory

The `inputs` directory contains files needed to run the pipeline.

```
hicseq.analysis-for-hicbench/inputs$
-rw-r--r--  1 at570   483 Dec 28 12:20 README
lrwxrwxrwx  1 at570     7 Oct  1 14:31 code -> ../code
lrwxrwxrwx  1 at570     7 Dec 21 08:19 data -> ../data
drwxr-xr-x  2 at570   685 Oct 27 13:46 fastq
lrwxrwxrwx  1 at570    12 Jan  5 09:23 genomes -> data/genomes
drwxr-xr-x  2 at570    29 Feb 15 13:30 params
lrwxrwxrwx  1 at570     5 Feb 11 15:24 results -> fastq
-rw-r--r--  1 at570  4.0K Feb 11 15:25 sample-sheet.tsv
```

- README: File containing usage notes for the inputs directory.
- code: Symlink to code one level up in the parent directory.
- data: Symlink to data one level up in the parent directory.
- fastq: Directory containing sub-directories for each sample to be used in the analysis. This directory is not created automatically, it must be created and populated manually. Alternatively, the directory bam can be used in its place if .bam files are to be used.
- genomes: Symlink to the directory containing reference genome information, within the data directory.
- params: Directory containing the parameters files associated with the input files.
- sample-sheet.tsv: Sample sheet for pipeline execution.

2.4.1 FASTQ Directory

The contents of an example fastq directory can be seen here:

```
hicseq.analysis-for-hicbench/inputs/fastq$
lrwxrwxrwx 1 at570  97 Feb 12 15:43 CD34-HindIII-rep1
lrwxrwxrwx 1 at570  95 Feb 12 15:43 GM-HindIII-rep1
lrwxrwxrwx 1 at570  92 Feb 12 15:43 GM-NcoI-rep1
lrwxrwxrwx 1 at570 103 Feb 12 15:43 H1-HindIII-Ren2015_rep1
lrwxrwxrwx 1 at570 103 Feb 12 15:43 H1-HindIII-Ren2015_rep2
lrwxrwxrwx 1 at570  95 Feb 12 15:43 H1-HindIII-rep1
lrwxrwxrwx 1 at570  95 Feb 12 15:43 H1-HindIII-rep2
lrwxrwxrwx 1 at570  98 Feb 12 15:43 IMR90-HindIII-rep1
lrwxrwxrwx 1 at570  98 Feb 12 15:43 IMR90-HindIII-rep2
lrwxrwxrwx 1 at570 100 Feb 12 15:43 T47D_T0-HindIII-rep1
lrwxrwxrwx 1 at570  97 Feb 12 15:43 T47D_T0-NcoI-rep1
lrwxrwxrwx 1 at570 101 Feb 12 15:43 T47D_T60-HindIII-rep1
lrwxrwxrwx 1 at570  98 Feb 12 15:43 T47D_T60-NcoI-rep1
lrwxrwxrwx 1 at570 100 Feb 12 15:43 mESC_J1-HindIII-rep1
lrwxrwxrwx 1 at570 100 Feb 12 15:43 mESC_J1-HindIII-rep2
lrwxrwxrwx 1 at570  97 Feb 12 15:43 mESC_J1-NcoI-rep1
```

Each directory name contains information about the sample, in the format <CellLine>-<treatment>-<SampleID>. This format can be modified to suit your purposes, though it is recommended to retain the "-" character as a delimiter since it is used downstream in the sample sheet generation steps. Each directory should contain all of the .fastq / .fastq.gz files associated with the sample; symlinks pointing to each file can be used as well, and are encouraged in order to save disk space. The same protocol should be followed if .bam files are to be used. As per standard Linux Terminal guidelines, spaces and special characters should be avoided in file names and directory names.

2.5 Pipeline Directory

The pipeline directory contains information for each step in the pipeline. An example pipeline directory will have the following structure:

```
hicseq.analysis-for-hicbench/pipeline$
drwxr-xr-x  5 at570 228 Feb 15 16:47 align
drwxr-xr-x  5 at570 207 Jan 19 22:12 annotations
drwxr-xr-x  5 at570 213 Feb 16 17:24 annotations-stats
drwxr-xr-x  5 at570 211 Feb  6 17:46 boundary-scores
drwxr-xr-x  5 at570 389 Mar 10 18:16 boundary-scores-pca
lrwxrwxrwx  1 at570   7 Dec  2 12:39 code -> ../code
lrwxrwxrwx  1 at570  12 Dec  2 12:39 code.main -> ../code.main
drwxr-xr-x  5 at570 385 Jan 19 22:08 compare-boundaries
drwxr-xr-x  5 at570 437 Mar 10 18:16 compare-boundaries-stats
```

```
drwxr-xr-x  5 at570 212 Jan 19 16:04 compare-matrices
drwxr-xr-x  5 at570 429 Mar 10 18:15 compare-matrices-stats
drwxr-xr-x  4 at570 420 Jan 19 22:10 diff-domains
drwxr-xr-x  5 at570 231 Jan 20 13:06 domains
drwxr-xr-x  5 at570 229 Jan 19 16:19 filter
drwxr-xr-x  6 at570 256 Jan 19 15:53 filter-stats
drwxr-xr-x  5 at570 206 Jan 19 22:11 hicplotter
-rw-r--r--  1 at570 331 Feb 14 19:28 index.txt
lrwxrwxrwx  1 at570   9 Dec  2 12:39 inputs -> ../inputs
drwxr-xr-x  5 at570 208 Jan 19 22:12 interactions
drwxr-xr-x  4 at570 362 Jan 19 16:01 matrix-estimated
drwxr-xr-x  5 at570 211 Jan 19 16:08 matrix-filtered
drwxr-xr-x  5 at570 794 Feb  7 17:05 matrix-hicnorm
drwxr-xr-x  5 at570 205 Jan 19 16:00 matrix-ic
drwxr-xr-x  5 at570 207 Jan 19 15:59 matrix-prep
drwxr-xr-x  5 at570 361 Jan 19 16:03 matrix-stats
drwxr-xr-x  4 at570 158 Dec 22 17:44 template
drwxr-xr-x  5 at570 229 Jan 19 15:55 tracks
```

- `align ... qc`: Directories containing the information for each pipeline step.
- `code`: Symlink to the directory containing code specific to current the analysis type.
- `code.main`: Symlink to the directory containing code used for all analyses.
- `inputs`: Symlink to the `inputs` directory containing the .fastq or .bam files for the pipeline.
- `index.txt`: A text file containing a list of pipeline steps to be executed. Entries in this document match the names of the pipeline directories.

2.5.1 Pipeline Index

The file `index.txt` contains a list of the pipeline steps to be completed during the analysis, listed in order of completion. An example `index.txt` would have the following structure:

```
hicseq.analysis-for-hicbench/pipeline$ cat index.txt
align

filter
filter-stats

tracks

matrix-filtered

matrix-prep

matrix-ic

matrix-hicnorm

#matrix-estimated
#
matrix-stats

compare-matrices
compare-matrices-stats

boundary-scores
boundary-scores-pca

domains

compare-boundaries
compare-boundaries-stats

#diff-domains
#
hicplotter

interactions
```

```
annotations
annotations-stats
```

Each entry in the `index.txt` file matches the name of the pipeline step to be completed, represented by the corresponding name of the step's sub-directory in the `pipeline` directory. One entry is allowed per line in the `index.txt` file. Entries that begin with a '#' character will be ignored, and pipeline steps that are not included in the `index.txt` file will not be included in the analysis pipeline.

2.5.2 Example Pipeline Step Directory Structure

Each step in the pipeline is represented by a sub-directory in the `pipeline` directory. An example sub-directory for a pipeline step would have the following structure:

```
hicseq.analysis-for-hicbench/pipeline/align$
lrwxrwxrwx  1 at570  15 Oct 28 12:10 clean.tcsh -> code/clean.tcsh
lrwxrwxrwx  1 at570   7 Sep 29 13:31 code -> ../code
-rw-r--r--  1 at570   0 Jan 25 11:12 error.log
drwxr-xr-x  2 at570  24 Feb 15 16:47 inpdirs
lrwxrwxrwx  1 at570   9 Sep 29 13:31 inputs -> ../inputs
drwxr-xr-x  2 at570  77 Dec 28 13:42 params
drwxr-xr-x  3 at570  62 Feb 16 12:27 results
lrwxrwxrwx  1 at570  14 Jan 19 15:50 run -> run-align.tcsh
-rwxr-xr-x  1 at570 971 Jan 19 15:49 run-align.tcsh
```

- `clean.tcsh`: Script for cleaning the directory; remove results and error logs.
- `code`: Symlink to the directory containing code specific to the analysis type e.g. `code.chipseq-standard` in this case.
- `error.log`: File containing errors encountered during execution of the pipeline step, generated at runtime.
- `inpdirs`: Directory containing symlinks to directories containing input files for use during execution of the pipeline step.
- `inputs`: Symlink to the directory containing input files.
- `params`: Directory containing the parameters files associated with the pipeline step files.
- `run`: Symlink to the 'run' file for the pipeline step.
- `run-align.tcsh`: 'Run' file for the pipeline step, containing a script that passes pipeline execution information to the wrapper script located in `./code/code.main/pipeline-master-explorer.r`.

2.5.3 Example Pipeline Step Results Directory

The base level of a results directory for a pipeline step will have the following structure:

```
hicseq.analysis-for-hicbench/pipeline/align/results/align.by_sample.bowtie2/CD34-HindIII-rep1$
-rw-r--r--  1 at570  49G Jan 13 01:02 alignments.bam
-rw-r--r--  1 at570  473 Jan 13 01:02 job.err
-rw-r--r--  1 at570   47 Jan 12 18:42 job.id
-rw-r--r--  1 at570    0 Jan 12 18:42 job.out
-rw-r--r--  1 at570  136 Jan 12 18:42 job.sh
-rw-r--r--  1 at570 2.3K Jan 13 01:02 job.vars.tsv
```

- `alignments.bam`: Example alignment output file.
- `job.err`: File containing the standard error output of the pipeline step.

- `job.id`: File containing the ID number of the job after submission for execution on the HPC cluster.
- `job.out`: File containing the standard output of the pipeline step.
- `job.sh`: File containing the command submitted for execution on the HPC cluster.
- `job.vars.tsv`: File containing the variables used in the completion of the pipeline step.

3 Adding Custom Pipeline Steps

3.1 Custom Pipeline Step Overview

The following basic steps should be taken to create a custom pipeline step:

- Copy an existing step as a template
- Update the new pipeline step name and add it to the entries in the `index.txt` and as a symlink in the parent level of the analysis directory
- Set the input directories ('inpdirs')
- Edit the 'run' file and add needed parameter files
- Add a script in the `code` directory containing the commands needed to run the programs used in the pipeline step

3.2 How To Add Custom Pipeline Steps

The steps needed to create a custom pipeline step are explained in detail here:

1. Within the `pipeline` directory, use a command such as `cp -r` to make a copy of an existing pipeline step as a template for the new one.

   Example `pipeline` directory:

```
hicseq.analysis-for-hicbench/pipeline$ ls -l
total 818K
drwxr-xr-x 25 at570 at570  834 Mar 18 17:14 .
drwxr-xr-x  5 at570 at570  958 Mar 21 19:09 ..
drwxr-xr-x  5 at570 at570  228 Mar 10 16:20 align
drwxr-xr-x  5 at570 at570  234 Mar 21 19:18 annotations
drwxr-xr-x  5 at570 at570  240 Mar 21 19:40 annotations-stats
drwxr-xr-x  5 at570 at570  238 Mar 18 20:44 boundary-scores
drwxr-xr-x  5 at570 at570  242 Mar 21 16:37 boundary-scores-pca
lrwxrwxrwx  1 at570 at570    7 Mar 10 16:20 code -> ../code
lrwxrwxrwx  1 at570 at570   12 Mar 10 16:20 code.main -> ../code.main
drwxr-xr-x  5 at570 at570  241 Mar 21 16:37 compare-boundaries
drwxr-xr-x  5 at570 at570  247 Mar 21 16:37 compare-boundaries-stats
drwxr-xr-x  5 at570 at570  239 Mar 18 20:20 compare-matrices
drwxr-xr-x  5 at570 at570  245 Mar 21 16:37 compare-matrices-stats
drwxr-xr-x  4 at570 at570  245 Mar 21 16:37 diff-domains
drwxr-xr-x  5 at570 at570  230 Mar 18 21:22 domains
drwxr-xr-x  5 at570 at570  229 Mar 10 16:20 filter
drwxr-xr-x  6 at570 at570  256 Mar 10 16:20 filter-stats
drwxr-xr-x  7 at570 at570  323 Mar 21 16:41 hicplotter
-rw-r--r--  1 at570 at570  331 Mar 10 16:20 index.txt
lrwxrwxrwx  1 at570 at570    9 Mar 10 16:20 inputs -> ../inputs
drwxr-xr-x  5 at570 at570  235 Mar 10 16:20 interactions
drwxr-xr-x  4 at570 at570  187 Mar 21 16:37 matrix-estimated
drwxr-xr-x  5 at570 at570  238 Mar 10 16:20 matrix-filtered
drwxr-xr-x  5 at570 at570  260 Mar 10 16:20 matrix-hicnorm
drwxr-xr-x  5 at570 at570  232 Mar 10 16:20 matrix-ic
drwxr-xr-x  5 at570 at570  234 Mar 18 17:31 matrix-prep
drwxr-xr-x  5 at570 at570  235 Mar 21 16:37 matrix-stats
lrwxrwxrwx  1 at570 hpchic    8 Dec  3 14:56 psync -> ../psync
drwxr-xr-x  4 at570 at570  158 Mar 10 16:20 template
drwxr-xr-x  5 at570 at570  229 Mar 10 16:20 tracks
```

2. Adjust the name of the new directory to match the desired name of the new pipeline step. Add this name as an entry in the `index.txt` file, and make a symlink to this directory from the parent directory in the same style of the existing symlinks to other pipeline steps. The alpha-numeric prefix on the symlink will determine the order in which it will be executed. The command to do this might look like this:

```
hicseq.analysis-for-hicbench$ ln -s pipeline/my_new_step __03b-my_new_step
```

Example `index.txt` file contents:

```
hicseq.analysis-for-hicbench/pipeline$ cat index.txt
align

filter
filter-stats

tracks

matrix-filtered

matrix-prep

matrix-ic

matrix-hicnorm

#matrix-estimated
#
matrix-stats

compare-matrices
compare-matrices-stats

boundary-scores
boundary-scores-pca

domains

compare-boundaries
compare-boundaries-stats

#diff-domains
#
hicplotter

interactions

annotations
annotations-stats
```

Example parent directory structure:

```
hicseq.analysis-for-hicbench$ ls -l
lrwxrwxrwx  1 at570 at570  14 Mar 10 16:20 __01a-align -> pipeline/align
lrwxrwxrwx  1 at570 at570  15 Mar 10 16:20 __02a-filter -> pipeline/filter
lrwxrwxrwx  1 at570 at570  21 Mar 10 16:20 __02b-filter-stats -> pipeline/filter-stats
lrwxrwxrwx  1 at570 at570  15 Mar 10 16:20 __03a-tracks -> pipeline/tracks
lrwxrwxrwx  1 at570 at570  24 Mar 10 16:20 __04a-matrix-filtered -> pipeline/matrix-filtered
lrwxrwxrwx  1 at570 at570  20 Mar 10 16:20 __05a-matrix-prep -> pipeline/matrix-prep
lrwxrwxrwx  1 at570 at570  18 Mar 10 16:20 __06a-matrix-ic -> pipeline/matrix-ic
lrwxrwxrwx  1 at570 at570  23 Mar 10 16:20 __07a-matrix-hicnorm -> pipeline/matrix-hicnorm
lrwxrwxrwx  1 at570 at570  21 Mar 10 16:20 __08a-matrix-stats -> pipeline/matrix-stats
lrwxrwxrwx  1 at570 at570  25 Mar 10 16:20 __09a-compare-matrices -> pipeline/compare-matrices
lrwxrwxrwx  1 at570 at570  31 Mar 10 16:20 __09b-compare-matrices-stats -> pipeline/compare-matrices-stats
lrwxrwxrwx  1 at570 at570  24 Mar 10 16:20 __10a-boundary-scores -> pipeline/boundary-scores
lrwxrwxrwx  1 at570 at570  28 Mar 10 16:20 __10b-boundary-scores-pca -> pipeline/boundary-scores-pca
lrwxrwxrwx  1 at570 at570  16 Mar 10 16:20 __11a-domains -> pipeline/domains
lrwxrwxrwx  1 at570 at570  27 Mar 10 16:20 __12a-compare-boundaries -> pipeline/compare-boundaries
lrwxrwxrwx  1 at570 at570  33 Mar 10 16:20 __12b-compare-boundaries-stats -> pipeline/compare-boundaries-stats
lrwxrwxrwx  1 at570 at570  19 Mar 10 16:20 __13a-hicplotter -> pipeline/hicplotter
lrwxrwxrwx  1 at570 at570  21 Mar 10 16:20 __14a-interactions -> pipeline/interactions
lrwxrwxrwx  1 at570 at570  20 Mar 10 16:20 __15a-annotations -> pipeline/annotations
lrwxrwxrwx  1 at570 at570  26 Mar 10 16:20 __15b-annotations-stats -> pipeline/annotations-stats
lrwxrwxrwx  1 at570 at570  30 Mar 14 18:25 code -> code.repo/code.hicseq-standard
lrwxrwxrwx  1 at570 at570  14 Mar 10 16:20 code.main -> code/code.main
drwxr-xr-x 10 at570 at570 238 Mar 13 21:45 code.repo
lrwxrwxrwx  1 at570 at570 104 Mar 14 18:25 data -> /ifs/home/at570/disk1/Resources/Code/pipeline-master/code/code.main/../../pipelines/hicseq-standard/data
drwxr-xr-x  5 at570 at570 274 Mar 10 16:20 inputs
```

```
drwxr-xr-x 25 at570 at570 834 Mar 18 17:14 pipeline
-rwxr-xr-x  1 at570 at570 211 Mar 11 12:06 psync
-rwxr-xr-x  1 at570 at570 888 Mar 10 16:20 run
-rwxr-xr-x  1 at570 at570 898 Mar 10 16:20 run.dry
```

3. Edit the contents of the directory you have created to hold the information for your new pipeline step.

   Example pipeline step directory:

```
hicseq.analysis-for-hicbench/pipeline/domains$ ls -l
lrwxrwxrwx 1 at570 at570  15 Mar 10 16:20 clean.tcsh -> code/clean.tcsh
lrwxrwxrwx 1 at570 at570   7 Mar 10 16:20 code -> ../code
-rw-r--r-- 1 at570 at570   0 Mar 18 21:22 error.log
drwxr-xr-x 2 at570 at570 155 Mar 10 16:20 inpdirs
lrwxrwxrwx 1 at570 at570   9 Mar 10 16:20 inputs -> ../inputs
drwxr-xr-x 3 at570 at570 146 Mar 10 16:20 params
drwxr-xr-x 6 at570 at570 161 Mar 18 20:48 results
lrwxrwxrwx 1 at570 at570  16 Mar 10 16:20 run -> run-domains.tcsh
-rwxr-xr-x 1 at570 at570 959 Mar 11 14:58 run-domains.tcsh
```

   First, edit the `run` file. A sample `run` file looks like this:

```
hicseq.analysis-for-hicbench/pipeline/domains$ cat run
#!/bin/tcsh
source ./code/code.main/custom-tcshrc      # customize shell environment

##
## USAGE: run-domains.tcsh [--dry-run]
##

# this section holds information that will be used in future updates of the software for reporting
#% This step identifies topologically-associated domains (TADs) using different methods.
#% TABLES:
#% FIGURES:

# process command-line inputs
# check to make sure that the proper number of arguments have been passed to the script,
# if not then print the script lines starting with '##' and exit
if ($#argv > 1) then
  grep '^##' $0 | scripts-send2err
  exit
endif

set opt = "$1"

# setup
# set the 'operation' to be performed, aka name of the pipeline step
set op = domains
# the directories to be used for inputs
set inpdirs = "inpdirs/*"
# an expression which specifies which input branches to include
set filter = "*.res_40kb"                  # work only with 40kb resolution
# the name of the results directory
set results = results

# create the results directory
scripts-create-path $results/
# sends a message to the error logging script
scripts-send2err "=== Operation = $op ============="
# 'resources' argument to be passed to qsub, referring to CPU cores and GB of RAM to be reserved for the
    job
set resources = 1,20G
# command to be passed to the 'pipeline-master-explorer.r' script
set cmd = "./code/code.main/scripts-qsub-wrapper $resources ./code/hicseq-$op.tcsh"

# generate run script
# the 'pipeline-master-explorer.r' script parses the items set above to create a line of text containing
    the commands to be submitted to qsub
Rscript ./code/code.main/pipeline-master-explorer.r -v -F "$filter" "$cmd" $results/$op "params/params.*.
    tcsh" "$inpdirs" "" "sample" 1

# run and wait until done!
# if the '--dry-run' argument was not passed to the script
if ("$opt" != "--dry-run") scripts-submit-jobs ./$results/.db/run
```

As listed in the above `run` file, the following 'setup' items need to be set for the custom pipeline step:

- ```
  set op = <name_of_pipeline_step>
  ```

  The 'operation' to be performed is set as 'op' and should be the name of the pipeline step, as listed in the directory name and in the `index.txt` file.

- ```
  set inpdirs = "inpdirs/*"
  ```

  The 'inpdirs', or input directories, should be set as the file path to the directory containing symlinks to the input directories. In this case, the contents of `inpdirs` is as follows:

  ```
  hicseq.analysis-for-hicbench/pipeline/domains$ ls -l inpdirs/
  lrwxrwxrwx 1 at570 at570 22 Mar 10 16:20 matrix-estimated -> ../../matrix-estimated
  lrwxrwxrwx 1 at570 at570 21 Mar 10 16:20 matrix-filtered -> ../../matrix-filtered
  lrwxrwxrwx 1 at570 at570 20 Mar 10 16:20 matrix-hicnorm -> ../../matrix-hicnorm
  lrwxrwxrwx 1 at570 at570 15 Mar 10 16:20 matrix-ic -> ../../matrix-ic
  lrwxrwxrwx 1 at570 at570 17 Mar 10 16:20 matrix-prep -> ../../matrix-prep
  ```

  The setting "inpdirs/*" will cause all input directories to be used. The entries in the `inpdirs` directory should be set as needed for the execution of the custom pipeline step.

- ```
  set filter = "*.res_40kb"
  ```

  The 'filter' setting to be used when parsing the 'branches' of the input directory results, for inclusion in the execution of the pipeline step. In this example, only input branches that match the pattern `"*.res_40kb"` will be included. In this example, the following input branches are available:

  ```
  hicseq.analysis-for-hicbench/pipeline/domains$ ls -l inpdirs/matrix-filtered/results/
  drwxr-xr-x 3 at570 at570 43 Mar 11 14:43 matrix-filtered.by_sample.res_1000kb
  drwxr-xr-x 3 at570 at570 43 Mar 11 14:44 matrix-filtered.by_sample.res_100kb
  drwxr-xr-x 3 at570 at570 43 Mar 11 14:44 matrix-filtered.by_sample.res_10kb.maxd_5Mb.rotate45
  drwxr-xr-x 3 at570 at570 43 Mar 11 14:45 matrix-filtered.by_sample.res_40kb
  ```

  Based on the given 'filter' setting, only the following branch will be included:

  ```
  drwxr-xr-x 3 at570 at570 43 Mar 11 14:45 matrix-filtered.by_sample.res_40kb
  ```

  This allows for the exclusion of unnecessary analysis branches.

- ```
  set resources = 1,20G
  ```

  This sets the number of computer resources to be reserved by `qsub`, listed as CPU cores and GB of RAM. If RAM is not a concern, only CPU cores need to be listed. A range of values can be used for CPU cores, such as `8-64`, though the utility of this depends on many factors related to your high-performance computing infrastructure and the specifics of the program being run; more cores may not necessarily speed up execution of the task at hand.

- ```
  set cmd = "./code/code.main/scripts-qsub-wrapper $resources ./code/hicseq-$op.tcsh"
  ```

  This line does not need to be modified by the user, but should be noted since it refers to the file in the `code` directory that will be created later and used to execute the program used in the pipeline step. Importantly, the entry `./code/hicseq-$op.tcsh` in this case refers to the file `./code/hicseq-domains.tcsh`.

- ```
  Rscript ./code/code.main/pipeline-master-explorer.r -v -F "$filter" "$cmd" $results/$op "params/
      params.*.tcsh" "$inpdirs" "" "sample" 1
  ```

  Since this calls the settings that have already been made, this line of the 'run' file does not need to be edited unless the grouping and splitting variables need to be changed. In this case, the command uses the following arguments (as per Section 5.3):

  ```
  pipeline-master-explorer.r [OPTIONS] SCRIPT OUTDIR-PREFIX PARAM-SCRIPTS INPUT-BRANCHES SPLIT-VARIABLE
      OUTPUT-OBJECT-VARIABLE TUPLES
  ```

  Importantly, the 'split-variable' and 'output-object-variable' come from the headings of columns used as grouping factors in the `inputs/sample-sheet.tsv` file for the analysis; custom grouping factors can be included in the sample sheet and used here. The output of the `pipeline-master-explorer.r` script is stored in the file `results/.db/run` which is created when the `run` file is executed (the command `./run--dry-run` can be used to generate this without running the commands). An example entry will look like this:

  ```
  hicseq.analysis-for-hicbench/pipeline/domains$ head -n 1 results/.db/run
  ./code/code.main/scripts-qsub-wrapper 1,20G ./code/hicseq-domains.tcsh results/domains.by_sample.
      armatus.gamma_0.5/matrix-prep.by_sample.scale/matrix-filtered.by_sample.res_40kb/filter.
      by_sample.standard/align.by_sample.bowtie2/CD34-HindIII-rep1 params/params.armatus.gamma_0.5.
      tcsh inpdirs/matrix-prep/results/matrix-prep.by_sample.scale/matrix-filtered.by_sample.res_40kb/
      filter.by_sample.standard/align.by_sample.bowtie2 'CD34-HindIII-rep1'
  ```

  During pipeline step execution, these lines will be submitted to `qsub` by the script `./code/code.main/scripts-qsub-wrapper`.

4. Next, the parameter files must be set for the new pipeline step. These files are contained in the `params` directory:

   ```
   hicseq.analysis-for-hicbench/pipeline/domains$ ls -l params/
   -rwxr-xr-x 1 at570 at570 131 Mar 10 16:20 params.armatus.gamma_0.5.tcsh
   -rwxr-xr-x 1 at570 at570 480 Mar 10 16:20 params.hicmatrix.tcsh
   -rwxr-xr-x 1 at570 at570 155 Mar 10 16:20 params.topdom.tcsh
   ```

   Importantly, files must use the following naming scheme: `params.<name>.tcsh`. All files included in the `params` directory following this naming scheme will be evaluated as a separate 'branch' for analysis. An example parameters file looks like this:

   ```
   hicseq.analysis-for-hicbench/pipeline/domains$ cat params/params.armatus.gamma_0.5.tcsh
   #!/bin/tcsh

   source ./inputs/params/params.tcsh

   set tool = armatus
   set chrom_excluded = 'chr[MY]'
   set armatus_params = "-g 0.5"
   ```

Settings that are specific to each analysis branch should be included in these files. Sub-directories in the `results` directory will be created for each entry in the `params` directory, as can be seen here:

```
hicseq.analysis-for-hicbench/pipeline/domains$ ls -l results/
drwxr-xr-x 7 at570 at570 246 Mar 18 20:48 domains.by_sample.armatus.gamma_0.5
drwxr-xr-x 7 at570 at570 246 Mar 18 20:48 domains.by_sample.hicmatrix
drwxr-xr-x 7 at570 at570 246 Mar 18 20:49 domains.by_sample.topdom
```

Note that in this case, the full output directory name comes from the following components: `<pipeline_step>.by_<output-object-variable>.<params_entry>`

5. A script containing the commands needed to run the desired program must be created and placed in the `code` directory, of which a partial list is shown below as an example:

```
hicseq.analysis-for-hicbench/pipeline/domains$ ls -l code/hic*
-rwxr-xr-x 1 at570 at570  26528 Dec  8 11:42 code/hic-matrix.o
-rwxr-xr-x 1 at570 at570 117660 Mar 18 15:25 code/hic-matrix.r
-rwxr-xr-x 1 at570 at570  24440 Dec  8 11:42 code/hic-matrix.so
-rwxr-xr-x 1 at570 at570   2471 Mar 10 16:20 code/hicnorm-cis.r
-rwxr-xr-x 1 at570 at570   2542 Mar 10 16:20 code/hicseq-align.tcsh
-rwxr-xr-x 1 at570 at570   2352 Mar 10 16:20 code/hicseq-annotate-tables.tcsh
-rwxr-xr-x 1 at570 at570   3463 Mar 21 19:28 code/hicseq-annotations-enrichments.r
-rwxr-xr-x 1 at570 at570   1547 Mar 21 18:54 code/hicseq-annotations-stats.tcsh
-rwxr-xr-x 1 at570 at570   1274 Mar 10 16:20 code/hicseq-annotations.tcsh
-rwxr-xr-x 1 at570 at570   2098 Mar 14 17:04 code/hicseq-boundary-scores-pca.tcsh
-rwxr-xr-x 1 at570 at570   1649 Mar 10 16:20 code/hicseq-boundary-scores.tcsh
-rwxr-xr-x 1 at570 at570   1440 Mar 10 16:20 code/hicseq-compare-boundaries-stats.tcsh
-rwxr-xr-x 1 at570 at570   3138 Mar 10 16:20 code/hicseq-compare-boundaries.tcsh
-rwxr-xr-x 1 at570 at570   1362 Mar 10 16:20 code/hicseq-compare-matrices-stats.tcsh
-rwxr-xr-x 1 at570 at570   1596 Mar 10 16:20 code/hicseq-compare-matrices.tcsh
-rwxr-xr-x 1 at570 at570   1902 Mar 10 16:20 code/hicseq-diff-domains.tcsh
```

Importantly, the primary script must follow this naming scheme: `hicseq-<pipeline_step>.tcsh`. In this example, this would correspond to `hicseq-domains.tcsh`. Pre-existing scripts can be used as a template to set up your custom script. This example script has the following contents:

```
hicseq.analysis-for-hicbench/pipeline/domains$ cat code/hicseq-domains.tcsh
#!/bin/tcsh
source ./code/code.main/custom-tcshrc     # shell settings

##
## USAGE: hicseq-domains.tcsh OUTPUT-DIR PARAM-SCRIPT BRANCH OBJECT(S)
##

if ($#argv != 4) then
  grep '^##' $0
  exit
endif

set outdir = $1
set params = $2
set branch = $3
set objects = ($4)

# read variables from input branch
source ./code/code.main/scripts-read-job-vars $branch "$objects" "genome genome_dir bin_size"

# run parameter script
source $params

# create path
scripts-create-path $outdir/

# -------------------------------------
# -----     MAIN CODE BELOW ------------
# -------------------------------------

# Run domains
if (($tool == armatus) || ($tool == di) || ($tool == topdom) || ($tool == caltads) || ($tool == hicmatrix)
    ) then
  ./code/hicseq-domains-$tool.tcsh $outdir $params $branch "$objects"
else
```

```
  scripts-send2err "Error: unknown domain caller tool $tool."
  exit 1
endif

# ---------------------------------------------------------
# -------    MAIN CODE ABOVE --------------------
# ---------------------------------------------------------

# save variables
source ./code/code.main/scripts-save-job-vars

# done
scripts-send2err "Done."
```

The preamble of the script should require little user intervention, while the bulk of the user's custom pipeline code should be inserted between the 'MAIN CODE' blocks specified within the document. For reference on how to structure your custom code, compare the 'USAGE' entry with the evaluated command to be passed to the script in the `results/.db/run` file. For convenience, the sample entry is repeated below:

```
hicseq.analysis-for-hicbench/pipeline/domains$ head -n 1 results/.db/run
./code/code.main/scripts-qsub-wrapper 1,20G ./code/hicseq-domains.tcsh results/domains.by_sample.armatus.
    gamma_0.5/matrix-prep.by_sample.scale/matrix-filtered.by_sample.res_40kb/filter.by_sample.standard/
    align.by_sample.bowtie2/CD34-HindIII-rep1 params/params.armatus.gamma_0.5.tcsh inpdirs/matrix-prep/
    results/matrix-prep.by_sample.scale/matrix-filtered.by_sample.res_40kb/filter.by_sample.standard/
    align.by_sample.bowtie2 'CD34-HindIII-rep1'
```

While this primary script should be in the .tcsh format, subsequent scripts in the user's preferred language can be called. They should follow the same naming conventions as shown in the `code` directory example above.

4 HiC-Seq Pipeline

4.1 Pipeline Steps

Within the parent directory of an analysis, the default pipeline steps are listed as symlinks, in alpha-numeric order starting with "__", as seen here:

```
lrwxrwxrwx  1 at570  14 Feb  7 17:06 __01a-align -> pipeline/align
lrwxrwxrwx  1 at570  15 Feb  7 17:06 __02a-filter -> pipeline/filter
lrwxrwxrwx  1 at570  21 Feb  7 17:06 __02b-filter-stats -> pipeline/filter-stats
lrwxrwxrwx  1 at570  15 Feb  7 17:06 __03a-tracks -> pipeline/tracks
lrwxrwxrwx  1 at570  24 Feb  7 17:06 __04a-matrix-filtered -> pipeline/matrix-filtered
lrwxrwxrwx  1 at570  20 Feb  7 17:06 __05a-matrix-prep -> pipeline/matrix-prep
lrwxrwxrwx  1 at570  18 Feb  7 17:06 __06a-matrix-ic -> pipeline/matrix-ic
lrwxrwxrwx  1 at570  23 Feb  7 17:06 __07a-matrix-hicnorm -> pipeline/matrix-hicnorm
lrwxrwxrwx  1 at570  21 Feb  7 17:06 __08a-matrix-stats -> pipeline/matrix-stats
lrwxrwxrwx  1 at570  25 Feb  7 17:06 __09a-compare-matrices -> pipeline/compare-matrices
lrwxrwxrwx  1 at570  31 Feb  7 17:06 __09b-compare-matrices-stats -> pipeline/compare-matrices-stats
lrwxrwxrwx  1 at570  24 Feb  7 17:06 __10a-boundary-scores -> pipeline/boundary-scores
lrwxrwxrwx  1 at570  28 Feb  7 17:06 __10b-boundary-scores-pca -> pipeline/boundary-scores-pca
lrwxrwxrwx  1 at570  16 Feb  7 17:06 __11a-domains -> pipeline/domains
lrwxrwxrwx  1 at570  27 Feb  7 17:06 __12a-compare-boundaries -> pipeline/compare-boundaries
lrwxrwxrwx  1 at570  33 Feb  7 17:06 __12b-compare-boundaries-stats -> pipeline/compare-boundaries-stats
lrwxrwxrwx  1 at570  19 Feb  7 17:06 __13a-hicplotter -> pipeline/hicplotter
lrwxrwxrwx  1 at570  21 Feb  7 17:06 __14a-interactions -> pipeline/interactions
lrwxrwxrwx  1 at570  20 Feb  7 17:06 __15a-annotations -> pipeline/annotations
lrwxrwxrwx  1 at570  30 Feb  8 17:47 code -> code.repo/code.hicseq-standard
lrwxrwxrwx  1 at570  14 Nov 12 11:55 code.main -> code/code.main
drwxr-xr-x  9 at570 209 Jan  9 10:16 code.repo
lrwxrwxrwx  1 at570 103 Feb  8 17:47 data -> /ifs/home/.../data
drwxr-xr-x  6 at570 258 Jan  5 09:24 inputs
drwxr-xr-x 24 at570 799 Feb  7 17:06 pipeline
-rwxr-xr-x  1 at570 210 Dec  2 14:23 psync
-rwxr-xr-x  1 at570 981 Jan  5 19:40 run
-rwxr-xr-x  1 at570 554 Dec 18 17:02 run.dry
-rwxr-xr-x  1 at570 988 Jan 24 14:42 run.subset
-rwxr-xr-x  1 at570 165 Dec 26 08:09 run.usage
```

This functions in informing the user of the order of pipeline steps. Each symlink points back to a directory in the pipeline directory for the corresponding pipeline step, as shown here:

```
pipeline$
total 814K
drwxr-xr-x  4 at570 203 Jan 19 15:50 align
drwxr-xr-x  5 at570 207 Jan 19 22:12 annotations
drwxr-xr-x  5 at570 387 Feb  6 17:46 boundary-scores
drwxr-xr-x  5 at570 243 Feb  7 14:17 boundary-scores-pca
lrwxrwxrwx  1 at570   7 Dec  2 12:39 code -> ../code
lrwxrwxrwx  1 at570  12 Dec  2 12:39 code.main -> ../code.main
drwxr-xr-x  5 at570 390 Jan 19 22:08 compare-boundaries
drwxr-xr-x  5 at570 396 Jan 20 10:59 compare-boundaries-stats
drwxr-xr-x  5 at570 435 Jan 19 16:04 compare-matrices
drwxr-xr-x  6 at570 419 Jan 19 16:05 compare-matrices-stats
drwxr-xr-x  5 at570 445 Jan 19 22:10 diff-domains
drwxr-xr-x  5 at570 379 Jan 20 13:06 domains
drwxr-xr-x  5 at570 229 Jan 19 16:19 filter
drwxr-xr-x  6 at570 256 Jan 19 15:53 filter-stats
drwxr-xr-x  5 at570 206 Jan 19 22:11 hicplotter
-rw-r--r--  1 at570 297 Feb  6 17:52 index.txt
lrwxrwxrwx  1 at570   9 Dec  2 12:39 inputs -> ../inputs
drwxr-xr-x  5 at570 208 Jan 19 22:12 interactions
drwxr-xr-x  5 at570 239 Jan 19 16:01 matrix-estimated
drwxr-xr-x  5 at570 238 Jan 19 16:08 matrix-filtered
drwxr-xr-x  5 at570 232 Jan 19 16:00 matrix-ic
drwxr-xr-x  5 at570 234 Jan 19 15:59 matrix-prep
drwxr-xr-x  5 at570 208 Jan 19 16:03 matrix-stats
lrwxrwxrwx  1 at570   8 Dec  3 14:56 psync -> ../psync
drwxr-xr-x  4 at570 158 Dec 22 17:44 template
drwxr-xr-x  5 at570 229 Jan 19 15:55 tracks
```

The pipeline directory contains files and symlinks needed for each step in the pipeline. The steps to be exectuted are defined in two ways:

1. A file called 'index.txt' lists the names of each step in the pipeline, in the order in which they will be completed. This file is located in the 'pipeline' directory.

2. A subdirectory within the 'pipeline' directory with the same name as its corresponding entry in the 'index.txt' file must be included to hold the parameters and commands to be run, and the results produced.

Index file:

```
pipeline/index.txt$
align

filter
filter-stats

tracks

matrix-filtered

matrix-prep

matrix-ic

#matrix-estimated
#
matrix-stats

compare-matrices
compare-matrices-stats

boundary-scores
boundary-scores-pca

domains

compare-boundaries
compare-boundaries-stats

#diff-domains
#
hicplotter

interactions

annotations
```

Steps listed in 'index.txt' which have been commented out (i.e. start with a # character) will not be included in the analysis. Custom pipeline steps can be easily included by adding the corresponding entry to the 'index.txt' and creating a subdirectory within the 'pipeline' directory.

For details on adding custom pipeline steps, see Section **??**

4.1.1 Default Parameters

These parameters are used by default across pipeline steps.

```
/inputs/params/params.tcsh$
#!/bin/tcsh

# load basic tools
module unload samtools
module unload java
module unload gcc
module unload python
module load samtools/1.2.1
module load bedtools/2.22.0
module load java/1.7
module load picard-tools

# load tools required for each step of the pipeline (this can be overriden in local param scripts)
module load bowtie2/2.2.6
module load armatus/2014-05-19
module load caltads/0.1.0
```

```
module load ghmm/0.9

# sample sheet file
set sheet = inputs/sample−sheet.tsv
```

4.1.2 Pipeline Step Execution Flowchart

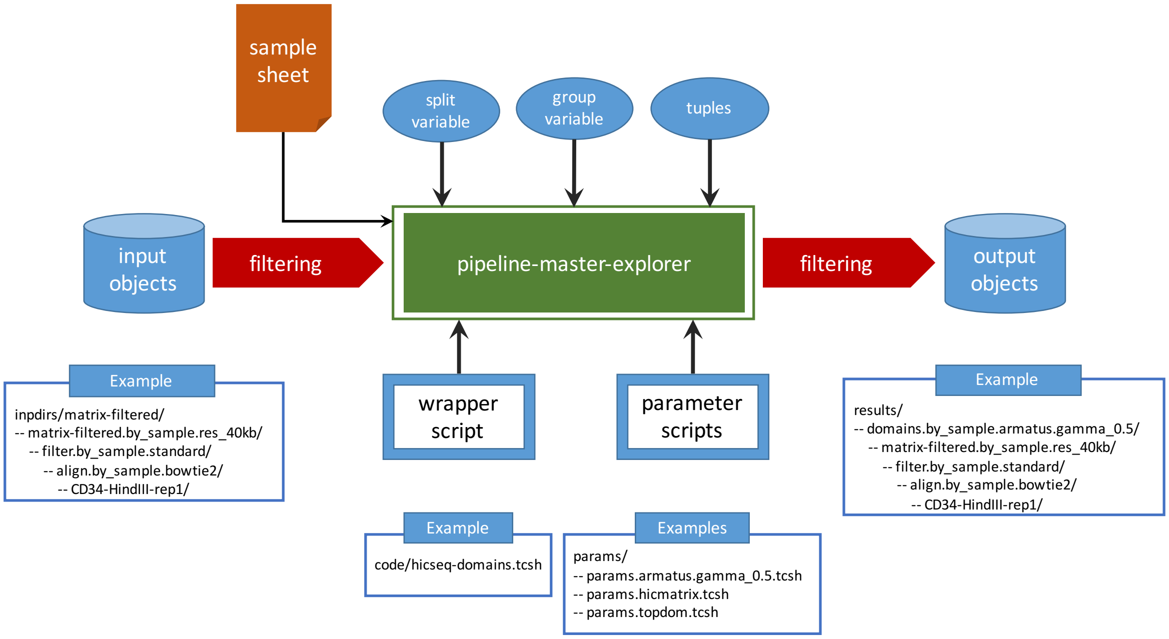

Figure 2: Overview of pipeline step execution for default analysis steps. See Section 4.1.2.

4.2 Alignment

4.2.1 Input

Raw data in fastq or fastq.gz files (Section 2.4).

4.2.2 Analysis

Default parameters:

```
params.bowtie2.tcsh$
#!/bin/tcsh

source ./inputs/params/params.tcsh

set aligner = bowtie2
set genome = `./code/read-sample-sheet.tcsh $sheet $object genome`
set genome_index = inputs/genomes/$genome/bowtie2.index/genome
set align_params = "--very-sensitive-local --local"
```

4.2.3 Output

Default output:

```
-rw-r--r-- 1 at570  49G Jan 13 01:02 alignments.bam
-rw-r--r-- 1 at570  473 Jan 13 01:02 job.err
-rw-r--r-- 1 at570   47 Jan 12 18:42 job.id
-rw-r--r-- 1 at570    0 Jan 12 18:42 job.out
-rw-r--r-- 1 at570  136 Jan 12 18:42 job.sh
-rw-r--r-- 1 at570 2.3K Jan 13 01:02 job.vars.tsv
```

4.3 Filter

4.3.1 Input

Data from the pipeline `align` step is used as input (Section 4.2).

4.3.2 Analysis

Default parameters:

```
params.standard.tcsh$
#!/bin/tcsh

source ./inputs/params/params.tcsh

set filter_params = "--mapq 30 --min-dist 25000 --max-offset 500 --filter-dups"
```

4.3.3 Output

Default output:

```
-rw-r--r--  1 at570 1.7G Jan 13 14:26 filtered.reg.gz
-rw-r--r--  1 at570  65K Jan 13 14:25 job.err
-rw-r--r--  1 at570   47 Jan 13 13:15 job.id
-rw-r--r--  1 at570    0 Jan 13 13:15 job.out
-rw-r--r--  1 at570  195 Jan 13 13:15 job.sh
-rw-r--r--  1 at570 2.1K Jan 13 14:26 job.vars.tsv
-rw-r--r--  1 at570  378 Jan 13 14:24 stats.tsv
```

4.4 Filter Stats

4.4.1 Input

Data from the pipeline `filter` step is used as input (Section 4.3).

4.4.2 Analysis

Default parameters:

```
params.standard.tcsh$
#!/bin/tcsh

source ./inputs/params/params.tcsh
```

4.4.3 Output

See Figure 3 and Figure 4. Default output:

```
-rw-r--r-- 1 at570 6.5K Feb 11 15:27 counts.pdf
-rw-r--r-- 1 at570   34 Feb 11 15:27 job.err
-rw-r--r-- 1 at570   47 Feb 11 15:27 job.id
-rw-r--r-- 1 at570   52 Feb 11 15:27 job.out
-rw-r--r-- 1 at570  226 Feb 11 15:27 job.sh
-rw-r--r-- 1 at570 6.7K Feb 11 15:27 percent.pdf
```

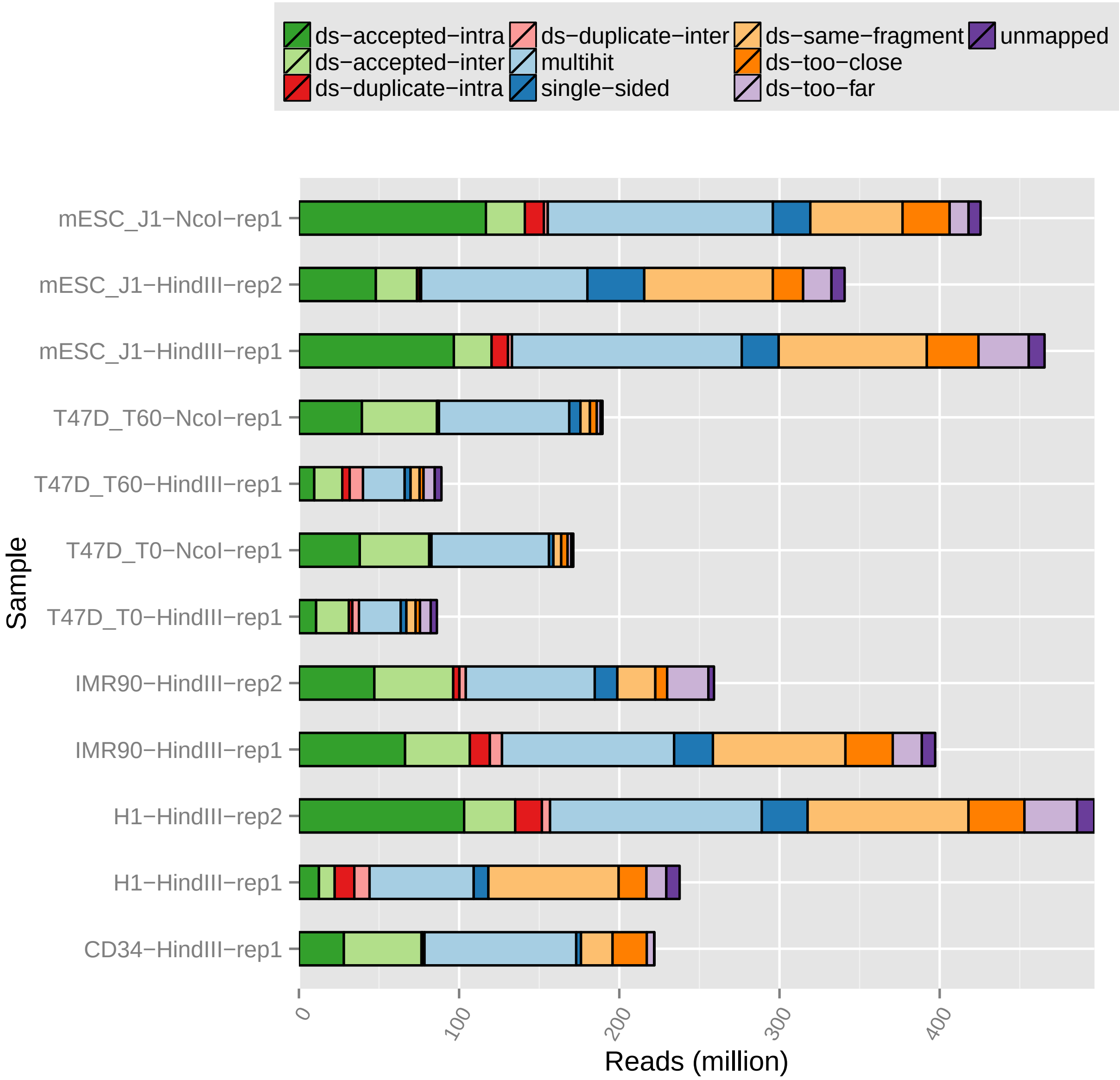

Figure 3: Filter Stats counts sample output

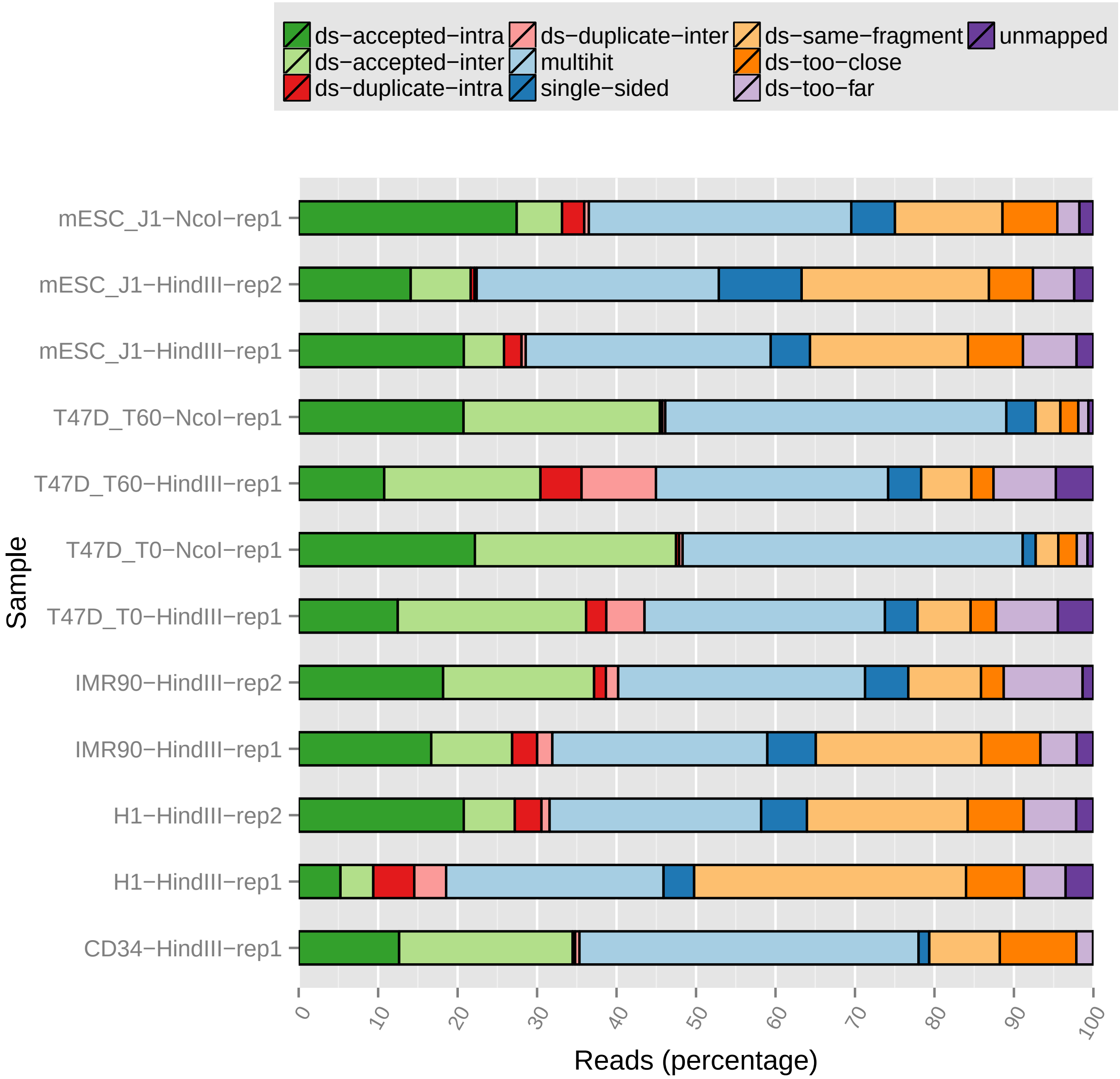

Figure 4: Filter Stats percentage sample output

4.5 Tracks

4.5.1 Input

Data from the pipeline `filter` step is used as input (Section 4.3).

4.5.2 Analysis

Default parameters:

```
params.standard.tcsh$
#!/bin/tcsh

source ./inputs/params/params.tcsh

set bin_size = 40000                # this is a commonly used bin size
```

4.5.3 Output

Default output:

```
-rw-r--r--  1 at570  4.1K Jan 13 15:55 job.err
-rw-r--r--  1 at570    47 Jan 13 15:10 job.id
-rw-r--r--  1 at570     0 Jan 13 15:11 job.out
-rw-r--r--  1 at570   242 Jan 13 15:10 job.sh
-rw-r--r--  1 at570  2.6K Jan 13 15:55 job.vars.tsv
-rw-r--r--  1 at570  1.1G Jan 13 15:54 track.washu.tsv.gz
-rw-r--r--  1 at570  789K Jan 13 15:55 track.washu.tsv.gz.tbi
```

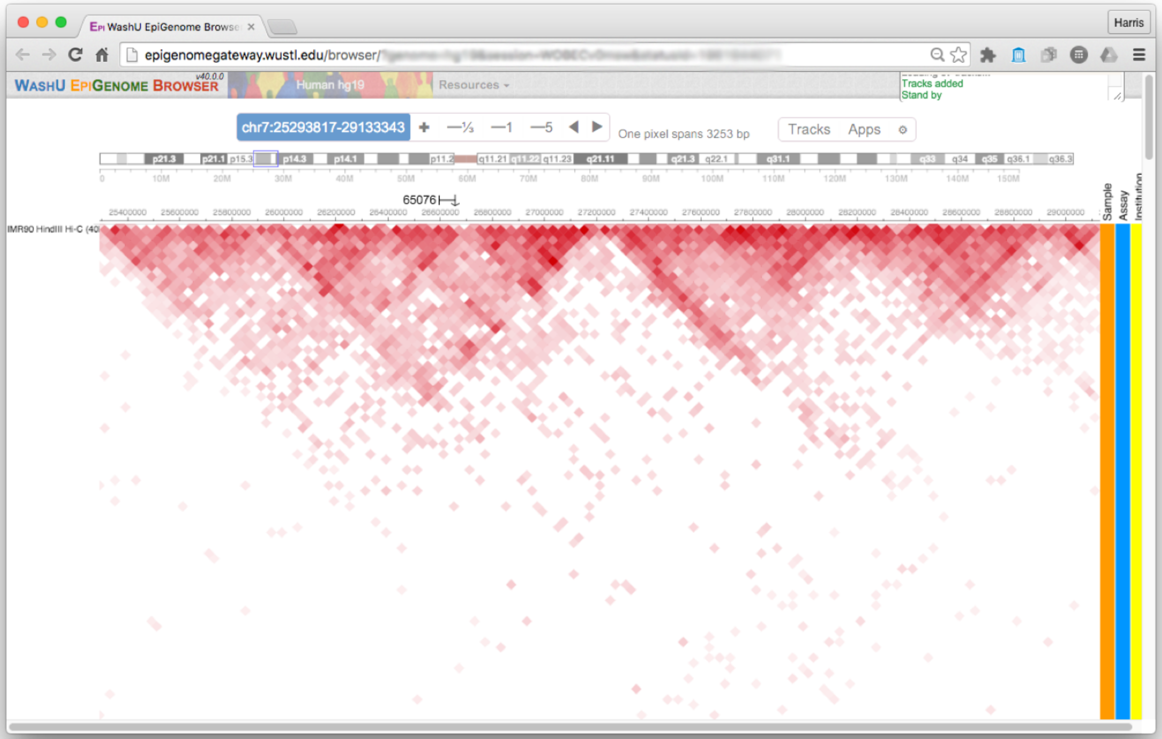

Figure 5: WashU tracks loaded in browser.

4.6 Matrix Filtered

4.6.1 Input

Data from the pipeline `filter` step is used as input (Section 4.3).

4.6.2 Analysis

Default parameters files:

```
-rwxr-xr-x 1 at570 195 Nov 24 11:20 params.res_1000kb.tcsh
-rwxr-xr-x 1 at570 194 Nov 25 15:11 params.res_100kb.tcsh
-rwxr-xr-x 1 at570 210 Dec  1 12:41 params.res_10kb.maxd_5Mb.rotate45.tcsh
-rwxr-xr-x 1 at570 193 Nov 30 16:22 params.res_10kb.tcsh
-rwxr-xr-x 1 at570 193 Nov 24 11:20 params.res_40kb.tcsh
```

Default parameters:

```
params.res_1000kb.tcsh$
#!/bin/tcsh

source ./inputs/params/params.tcsh

set bin_size = 1000000
set max_dist = 0
set ref = $genome_dir/genome.bed
set matrix_params = "--bin-size $bin_size --max-dist $max_dist -R $ref"
```

```
params.res_100kb.tcsh$
#!/bin/tcsh

source ./inputs/params/params.tcsh

set bin_size = 100000
set max_dist = 0
set ref = $genome_dir/genome.bed
set matrix_params = "--bin-size $bin_size --max-dist $max_dist -R $ref"
```

```
params.res_10kb.maxd_5Mb.rotate45.tcsh$
#!/bin/tcsh

source ./inputs/params/params.tcsh

set bin_size = 10000
set max_dist = 5000000
set ref = $genome_dir/genome.bed
set matrix_params = "--bin-size $bin_size --max-dist $max_dist --rotate45 -R $ref"
```

```
params.res_10kb.tcsh
#!/bin/tcsh

source ./inputs/params/params.tcsh

set bin_size = 10000
set max_dist = 0
set ref = $genome_dir/genome.bed
set matrix_params = "--bin-size $bin_size --max-dist $max_dist -R $ref"
```

```
params.res_40kb.tcsh$
#!/bin/tcsh

source ./inputs/params/params.tcsh
```

```
set bin_size = 40000
set max_dist = 0
set ref = $genome_dir/genome.bed
set matrix_params = "--bin-size $bin_size --max-dist $max_dist -R $ref"
```

4.6.3 Output

Default output:

```
-rw-r--r-- 1 at570  56K Jan 13 15:57 ignored_loci.txt
-rw-r--r-- 1 at570 9.6K Jan 13 16:02 job.err
-rw-r--r-- 1 at570   47 Jan 13 15:57 job.id
-rw-r--r-- 1 at570    0 Jan 13 15:57 job.out
-rw-r--r-- 1 at570  266 Jan 13 15:57 job.sh
-rw-r--r-- 1 at570 2.7K Jan 13 16:02 job.vars.tsv
-rw-r--r-- 1 at570  75M Jan 13 16:01 matrix.chr1.tsv
-rw-r--r-- 1 at570  23M Jan 13 16:01 matrix.chr10.tsv
-rw-r--r-- 1 at570  22M Jan 13 16:01 matrix.chr11.tsv
-rw-r--r-- 1 at570  22M Jan 13 16:01 matrix.chr12.tsv
-rw-r--r-- 1 at570  16M Jan 13 16:01 matrix.chr13.tsv
-rw-r--r-- 1 at570  14M Jan 13 16:01 matrix.chr14.tsv
-rw-r--r-- 1 at570  13M Jan 13 16:01 matrix.chr15.tsv
-rw-r--r-- 1 at570 9.9M Jan 13 16:01 matrix.chr16.tsv
-rw-r--r-- 1 at570 8.0M Jan 13 16:01 matrix.chr17.tsv
-rw-r--r-- 1 at570 7.4M Jan 13 16:01 matrix.chr18.tsv
-rw-r--r-- 1 at570 4.3M Jan 13 16:01 matrix.chr19.tsv
-rw-r--r-- 1 at570  71M Jan 13 16:01 matrix.chr2.tsv
-rw-r--r-- 1 at570 4.9M Jan 13 16:01 matrix.chr20.tsv
-rw-r--r-- 1 at570 2.9M Jan 13 16:01 matrix.chr21.tsv
-rw-r--r-- 1 at570 3.3M Jan 13 16:01 matrix.chr22.tsv
-rw-r--r-- 1 at570  48M Jan 13 16:01 matrix.chr3.tsv
-rw-r--r-- 1 at570  44M Jan 13 16:01 matrix.chr4.tsv
-rw-r--r-- 1 at570  40M Jan 13 16:01 matrix.chr5.tsv
-rw-r--r-- 1 at570  36M Jan 13 16:02 matrix.chr6.tsv
-rw-r--r-- 1 at570  31M Jan 13 16:02 matrix.chr7.tsv
-rw-r--r-- 1 at570  26M Jan 13 16:02 matrix.chr8.tsv
-rw-r--r-- 1 at570  24M Jan 13 16:02 matrix.chr9.tsv
-rw-r--r-- 1 at570   29 Jan 13 16:02 matrix.chrM.tsv
-rw-r--r-- 1 at570  29M Jan 13 16:02 matrix.chrX.tsv
-rw-r--r-- 1 at570 4.3M Jan 13 16:02 matrix.chrY.tsv
```

4.7 Matrix Prep

4.7.1 Input

Data from the pipeline `matrix-filtered` step is used as input (Section 4.6).

4.7.2 Analysis

Default parameters:

```
params.scale_impute.tcsh$
#!/bin/tcsh

source ./inputs/params/params.tcsh

set chrom_excluded = 'chr[MY]'       # excluded chromosomes
set prep_params = "--scale --impute"
```

4.7.3 Output

Default output:

```
drwxr-xr-x  2 at570 3.4K Jan 13 16:16 __jdata
-rw-r--r--  1 at570 4.0K Jan 13 16:18 job.err
-rw-r--r--  1 at570   47 Jan 13 16:14 job.id
-rw-r--r--  1 at570    0 Jan 13 16:15 job.out
-rw-r--r--  1 at570  345 Jan 13 16:14 job.sh
-rw-r--r--  1 at570 3.3K Jan 13 16:18 job.vars.tsv
-rw-r--r--  1 at570 371M Jan 13 16:17 matrix.chr1.tsv
-rw-r--r--  1 at570 110M Jan 13 16:16 matrix.chr10.tsv
-rw-r--r--  1 at570 109M Jan 13 16:15 matrix.chr11.tsv
-rw-r--r--  1 at570 107M Jan 13 16:16 matrix.chr12.tsv
-rw-r--r--  1 at570  80M Jan 13 16:16 matrix.chr13.tsv
-rw-r--r--  1 at570  69M Jan 13 16:16 matrix.chr14.tsv
-rw-r--r--  1 at570  63M Jan 13 16:16 matrix.chr15.tsv
-rw-r--r--  1 at570  49M Jan 13 16:16 matrix.chr16.tsv
-rw-r--r--  1 at570  40M Jan 13 16:16 matrix.chr17.tsv
-rw-r--r--  1 at570  37M Jan 13 16:16 matrix.chr18.tsv
-rw-r--r--  1 at570  21M Jan 13 16:16 matrix.chr19.tsv
-rw-r--r--  1 at570 353M Jan 13 16:17 matrix.chr2.tsv
-rw-r--r--  1 at570  24M Jan 13 16:16 matrix.chr20.tsv
-rw-r--r--  1 at570  14M Jan 13 16:16 matrix.chr21.tsv
-rw-r--r--  1 at570  16M Jan 13 16:16 matrix.chr22.tsv
-rw-r--r--  1 at570 234M Jan 13 16:17 matrix.chr3.tsv
-rw-r--r--  1 at570 219M Jan 13 16:17 matrix.chr4.tsv
-rw-r--r--  1 at570 196M Jan 13 16:17 matrix.chr5.tsv
-rw-r--r--  1 at570 175M Jan 13 16:17 matrix.chr6.tsv
-rw-r--r--  1 at570 152M Jan 13 16:17 matrix.chr7.tsv
-rw-r--r--  1 at570 128M Jan 13 16:17 matrix.chr8.tsv
-rw-r--r--  1 at570 120M Jan 13 16:17 matrix.chr9.tsv
-rw-r--r--  1 at570 144M Jan 13 16:17 matrix.chrX.tsv
```

4.8 Matrix IC

4.8.1 Input

Data from the pipeline `matrix-filtered` step is used as input (Section 4.6).

4.8.2 Analysis

Default parameters:

```
params.standard.tcsh$
#!/bin/tcsh

source ./inputs/params/params.tcsh

module unload gcc                # this is necessary in order to take care of module conflicts in our system
module unload python
module load python/2.7.3

set chrom_excluded = 'chr[MY]'        # excluded chromosomes
set cutoff = 0.05
```

4.8.3 Output

Default output:

```
drwxr-xr-x  2 at570 3.4K Jan 13 16:50 __jdata
-rw-r--r--  1 at570 1.4K Jan 13 16:52 job.err
-rw-r--r--  1 at570   47 Jan 13 16:47 job.id
-rw-r--r--  1 at570    0 Jan 13 16:47 job.out
-rw-r--r--  1 at570  333 Jan 13 16:47 job.sh
-rw-r--r--  1 at570 3.5K Jan 13 16:52 job.vars.tsv
-rw-r--r--  1 at570 371M Jan 13 16:50 matrix.chr1.tsv
-rw-r--r--  1 at570 110M Jan 13 16:49 matrix.chr10.tsv
-rw-r--r--  1 at570 109M Jan 13 16:49 matrix.chr11.tsv
-rw-r--r--  1 at570 107M Jan 13 16:49 matrix.chr12.tsv
-rw-r--r--  1 at570  80M Jan 13 16:49 matrix.chr13.tsv
-rw-r--r--  1 at570  69M Jan 13 16:49 matrix.chr14.tsv
-rw-r--r--  1 at570  63M Jan 13 16:49 matrix.chr15.tsv
-rw-r--r--  1 at570  49M Jan 13 16:49 matrix.chr16.tsv
-rw-r--r--  1 at570  40M Jan 13 16:49 matrix.chr17.tsv
-rw-r--r--  1 at570  37M Jan 13 16:49 matrix.chr18.tsv
-rw-r--r--  1 at570  21M Jan 13 16:49 matrix.chr19.tsv
-rw-r--r--  1 at570 353M Jan 13 16:52 matrix.chr2.tsv
-rw-r--r--  1 at570  24M Jan 13 16:49 matrix.chr20.tsv
-rw-r--r--  1 at570  14M Jan 13 16:50 matrix.chr21.tsv
-rw-r--r--  1 at570  16M Jan 13 16:50 matrix.chr22.tsv
-rw-r--r--  1 at570 234M Jan 13 16:51 matrix.chr3.tsv
-rw-r--r--  1 at570 219M Jan 13 16:51 matrix.chr4.tsv
-rw-r--r--  1 at570 196M Jan 13 16:51 matrix.chr5.tsv
-rw-r--r--  1 at570 175M Jan 13 16:51 matrix.chr6.tsv
-rw-r--r--  1 at570 152M Jan 13 16:51 matrix.chr7.tsv
-rw-r--r--  1 at570 128M Jan 13 16:51 matrix.chr8.tsv
-rw-r--r--  1 at570 120M Jan 13 16:51 matrix.chr9.tsv
-rw-r--r--  1 at570 144M Jan 13 16:51 matrix.chrX.tsv
```

4.9 Matrix HiCNorm

4.9.1 Input

Data from the pipeline `matrix-filtered` step is used as input (Section 4.6).

4.9.2 Analysis

Default parameters:

```
params.standard.tcsh$
#!/bin/tcsh

source ./inputs/params/params.tcsh

set chrom_excluded = 'chr[MY]'        # excluded chromosomes
```

4.9.3 Output

Default output:

```
drwxr-xr-x  2 at570 3.4K Feb  8 17:10 __jdata
-rw-r--r--  1 at570  13K Feb  8 17:25 job.err
-rw-r--r--  1 at570   47 Feb  8 17:06 job.id
-rw-r--r--  1 at570    0 Feb  8 17:06 job.out
-rw-r--r--  1 at570  343 Feb  8 17:06 job.sh
-rw-r--r--  1 at570 3.2K Feb  8 17:25 job.vars.tsv
-rw-r--r--  1 at570  86M Feb  8 17:19 matrix.chr1.tsv
-rw-r--r--  1 at570  28M Feb  8 17:10 matrix.chr10.tsv
-rw-r--r--  1 at570  28M Feb  8 17:12 matrix.chr11.tsv
-rw-r--r--  1 at570  28M Feb  8 17:10 matrix.chr12.tsv
-rw-r--r--  1 at570  21M Feb  8 17:09 matrix.chr13.tsv
-rw-r--r--  1 at570  18M Feb  8 17:09 matrix.chr14.tsv
-rw-r--r--  1 at570  16M Feb  8 17:09 matrix.chr15.tsv
-rw-r--r--  1 at570  13M Feb  8 17:09 matrix.chr16.tsv
-rw-r--r--  1 at570 9.7M Feb  8 17:09 matrix.chr17.tsv
-rw-r--r--  1 at570  11M Feb  8 17:09 matrix.chr18.tsv
-rw-r--r--  1 at570 5.3M Feb  8 17:08 matrix.chr19.tsv
-rw-r--r--  1 at570  85M Feb  8 17:25 matrix.chr2.tsv
-rw-r--r--  1 at570 6.6M Feb  8 17:10 matrix.chr20.tsv
-rw-r--r--  1 at570 3.6M Feb  8 17:09 matrix.chr21.tsv
-rw-r--r--  1 at570 3.8M Feb  8 17:09 matrix.chr22.tsv
-rw-r--r--  1 at570  58M Feb  8 17:17 matrix.chr3.tsv
-rw-r--r--  1 at570  55M Feb  8 17:20 matrix.chr4.tsv
-rw-r--r--  1 at570  49M Feb  8 17:15 matrix.chr5.tsv
-rw-r--r--  1 at570  44M Feb  8 17:15 matrix.chr6.tsv
-rw-r--r--  1 at570  38M Feb  8 17:17 matrix.chr7.tsv
-rw-r--r--  1 at570  33M Feb  8 17:14 matrix.chr8.tsv
-rw-r--r--  1 at570  29M Feb  8 17:13 matrix.chr9.tsv
-rw-r--r--  1 at570  34M Feb  8 17:15 matrix.chrX.tsv
```

4.10 Matrix Stats

4.10.1 Input

Data from the pipeline steps `matrix-filtered` (Section 4.6), `matrix-hicnorm` (Section 4.9), `matrix-prep` (Section 4.7), and `matrix-ic` (Section 4.8) are used as input.

4.10.2 Analysis

Default parameters:

```
params.standard.tcsh$
#!/bin/tcsh

source ./inputs/params/params.tcsh

set chrom_excluded = 'chr[MY]'                # excluded chromosomes
```

4.10.3 Output

See Figure 6. Default output:

```
-rw-r--r-- 1 at570  39K Feb 11 16:11 job.err
-rw-r--r-- 1 at570   47 Feb 11 15:48 job.id
-rw-r--r-- 1 at570    0 Feb 11 15:48 job.out
-rw-r--r-- 1 at570  480 Feb 11 15:48 job.sh
-rw-r--r-- 1 at570 5.3K Feb 11 16:11 job.vars.tsv
-rw-r--r-- 1 at570  59K Feb 11 16:11 stats.pdf
```

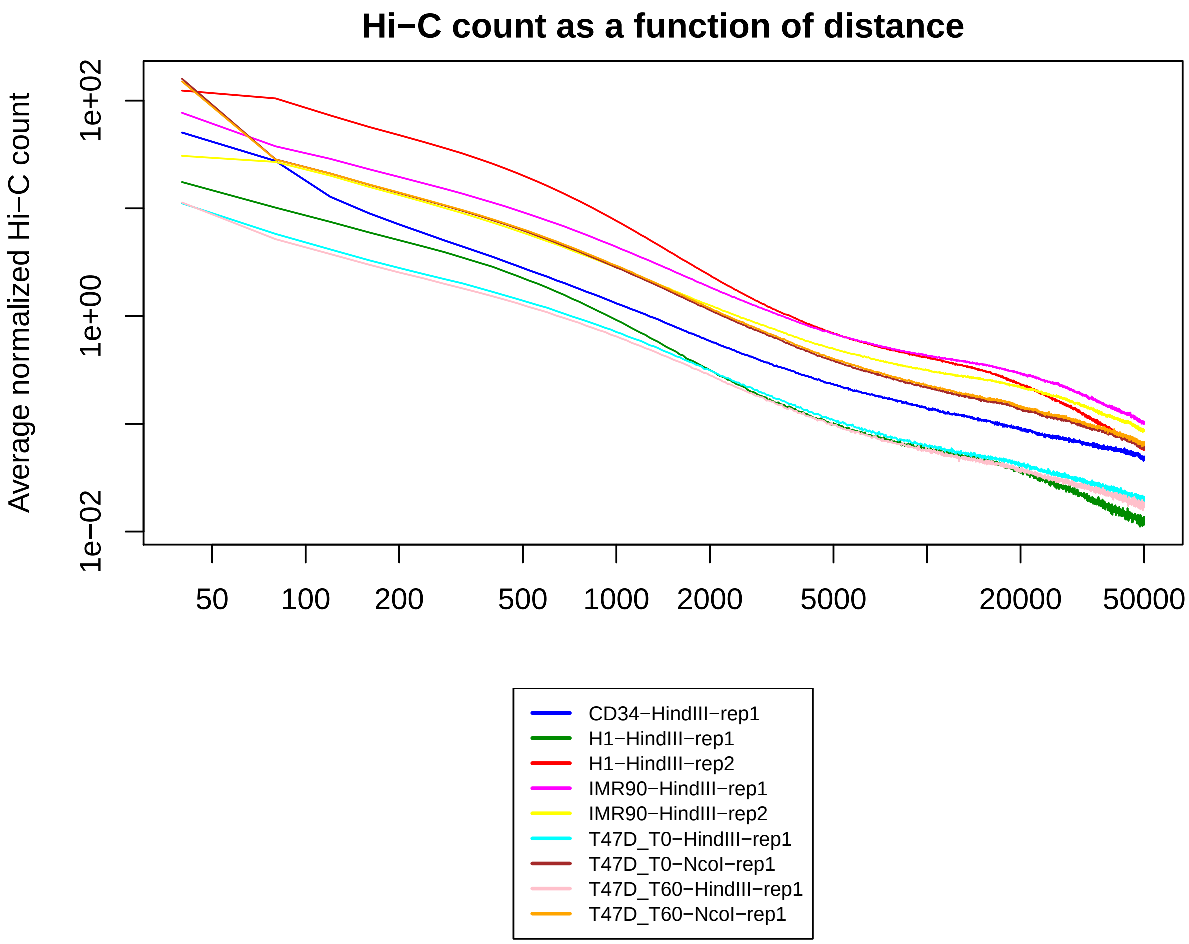

Figure 6: Matrix Stats sample output

4.11 Compare Matrices

4.11.1 Input

Data from the pipeline steps `matrix-filtered` (Section 4.6), `matrix-hicnorm` (Section 4.9), `matrix-prep` (Section 4.7), and `matrix-ic` (Section 4.8) are used as input.

4.11.2 Analysis

Default parameters:

```
params.standard.tcsh$
#!/bin/tcsh

source ./inputs/params/params.tcsh

set chrom_excluded = 'chr[MYX]'       # excluded chromosomes

set max_dist = `echo 10000000/$bin_size | bc`      # number of bins (max distance = 10Mb)

set compare_params = "--max-dist=$max_dist --n-dist=1 --min-lambda=0.0 --max-lambda=1.0 --n-lambda=6 --gamma=0"
              # only used if estimation was done with max-lambda=Inf
```

4.11.3 Output

Default output:

```
-rw-r--r--   1 at570   17 Feb  9 01:26 chr1.cor.pearson.tsv
-rw-r--r--   1 at570   17 Feb  9 01:26 chr1.cor.spearman.tsv
-rw-r--r--   1 at570   17 Feb  9 01:27 chr10.cor.pearson.tsv
-rw-r--r--   1 at570   17 Feb  9 01:27 chr10.cor.spearman.tsv
-rw-r--r--   1 at570   17 Feb  9 01:28 chr11.cor.pearson.tsv
-rw-r--r--   1 at570   17 Feb  9 01:28 chr11.cor.spearman.tsv
-rw-r--r--   1 at570   17 Feb  9 01:28 chr12.cor.pearson.tsv
-rw-r--r--   1 at570   17 Feb  9 01:28 chr12.cor.spearman.tsv
-rw-r--r--   1 at570   17 Feb  9 01:29 chr13.cor.pearson.tsv
-rw-r--r--   1 at570   17 Feb  9 01:29 chr13.cor.spearman.tsv
-rw-r--r--   1 at570   17 Feb  9 01:29 chr14.cor.pearson.tsv
-rw-r--r--   1 at570   17 Feb  9 01:29 chr14.cor.spearman.tsv
-rw-r--r--   1 at570   17 Feb  9 01:30 chr15.cor.pearson.tsv
-rw-r--r--   1 at570   17 Feb  9 01:30 chr15.cor.spearman.tsv
-rw-r--r--   1 at570   17 Feb  9 01:30 chr16.cor.pearson.tsv
-rw-r--r--   1 at570   17 Feb  9 01:30 chr16.cor.spearman.tsv
-rw-r--r--   1 at570   17 Feb  9 01:31 chr17.cor.pearson.tsv
-rw-r--r--   1 at570   17 Feb  9 01:31 chr17.cor.spearman.tsv
-rw-r--r--   1 at570   17 Feb  9 01:31 chr18.cor.pearson.tsv
-rw-r--r--   1 at570   17 Feb  9 01:31 chr18.cor.spearman.tsv
-rw-r--r--   1 at570   17 Feb  9 01:31 chr19.cor.pearson.tsv
-rw-r--r--   1 at570   17 Feb  9 01:31 chr19.cor.spearman.tsv
-rw-r--r--   1 at570   17 Feb  9 01:33 chr2.cor.pearson.tsv
-rw-r--r--   1 at570   17 Feb  9 01:33 chr2.cor.spearman.tsv
-rw-r--r--   1 at570   17 Feb  9 01:33 chr20.cor.pearson.tsv
-rw-r--r--   1 at570   17 Feb  9 01:33 chr20.cor.spearman.tsv
-rw-r--r--   1 at570   17 Feb  9 01:33 chr21.cor.pearson.tsv
-rw-r--r--   1 at570   17 Feb  9 01:33 chr21.cor.spearman.tsv
-rw-r--r--   1 at570   17 Feb  9 01:33 chr22.cor.pearson.tsv
-rw-r--r--   1 at570   17 Feb  9 01:33 chr22.cor.spearman.tsv
-rw-r--r--   1 at570   17 Feb  9 01:35 chr3.cor.pearson.tsv
-rw-r--r--   1 at570   17 Feb  9 01:35 chr3.cor.spearman.tsv
-rw-r--r--   1 at570   17 Feb  9 01:36 chr4.cor.pearson.tsv
-rw-r--r--   1 at570   17 Feb  9 01:36 chr4.cor.spearman.tsv
-rw-r--r--   1 at570   17 Feb  9 01:37 chr5.cor.pearson.tsv
-rw-r--r--   1 at570   17 Feb  9 01:37 chr5.cor.spearman.tsv
-rw-r--r--   1 at570   17 Feb  9 01:38 chr6.cor.pearson.tsv
-rw-r--r--   1 at570   17 Feb  9 01:38 chr6.cor.spearman.tsv
-rw-r--r--   1 at570   17 Feb  9 01:39 chr7.cor.pearson.tsv
-rw-r--r--   1 at570   17 Feb  9 01:39 chr7.cor.spearman.tsv
-rw-r--r--   1 at570   17 Feb  9 01:40 chr8.cor.pearson.tsv
-rw-r--r--   1 at570   17 Feb  9 01:40 chr8.cor.spearman.tsv
-rw-r--r--   1 at570   17 Feb  9 01:41 chr9.cor.pearson.tsv
-rw-r--r--   1 at570   17 Feb  9 01:41 chr9.cor.spearman.tsv
-rw-r--r--   1 at570 1.9K Feb  9 01:41 cor.pearson.tsv
```

```
-rw-r--r--   1 at570  1.9K Feb  9 01:41 cor.spearman.tsv
-rw-r--r--   1 at570  1.8K Feb  9 01:41 job.err
-rw-r--r--   1 at570    47 Feb  8 18:22 job.id
-rw-r--r--   1 at570     0 Feb  9 01:25 job.out
-rw-r--r--   1 at570   388 Feb  8 18:22 job.sh
-rw-r--r--   1 at570  3.1K Feb  9 01:41 job.vars.tsv
```

4.12 Compare Matrices Stats

4.12.1 Input

Data from the pipeline `compare-matrices` step is used as input (Section 4.11).

4.12.2 Analysis

Default parameters:

```
params.standard.tcsh$
#!/bin/tcsh

source ./inputs/params/params.tcsh
```

4.12.3 Output

See Figure 7, and See Figure 8. Default output:

```
-rw-r--r-- 1 at570   97 Feb 12 11:25 job.err
-rw-r--r-- 1 at570   47 Feb 12 11:24 job.id
-rw-r--r-- 1 at570   52 Feb 12 11:25 job.out
-rw-r--r-- 1 at570 3.4K Feb 12 11:24 job.sh
-rw-r--r-- 1 at570  44K Feb 12 11:25 job.vars.tsv
drwxr-xr-x 2 at570   96 Feb 12 11:25 pearson
drwxr-xr-x 2 at570   97 Feb 12 11:25 spearman
```

```
spearman/$
-rw-r--r-- 1 at570 161K Feb 12 11:25 cor.spearman.tsv
-rw-r--r-- 1 at570 8.9K Feb 12 11:25 correlograms.pdf
-rw-r--r-- 1 at570  12K Feb 12 11:25 summary.tsv
```

```
pearson/$
-rw-r--r-- 1 at570 159K Feb 12 11:25 cor.pearson.tsv
-rw-r--r-- 1 at570 9.0K Feb 12 11:25 correlograms.pdf
-rw-r--r-- 1 at570  12K Feb 12 11:25 summary.tsv
```

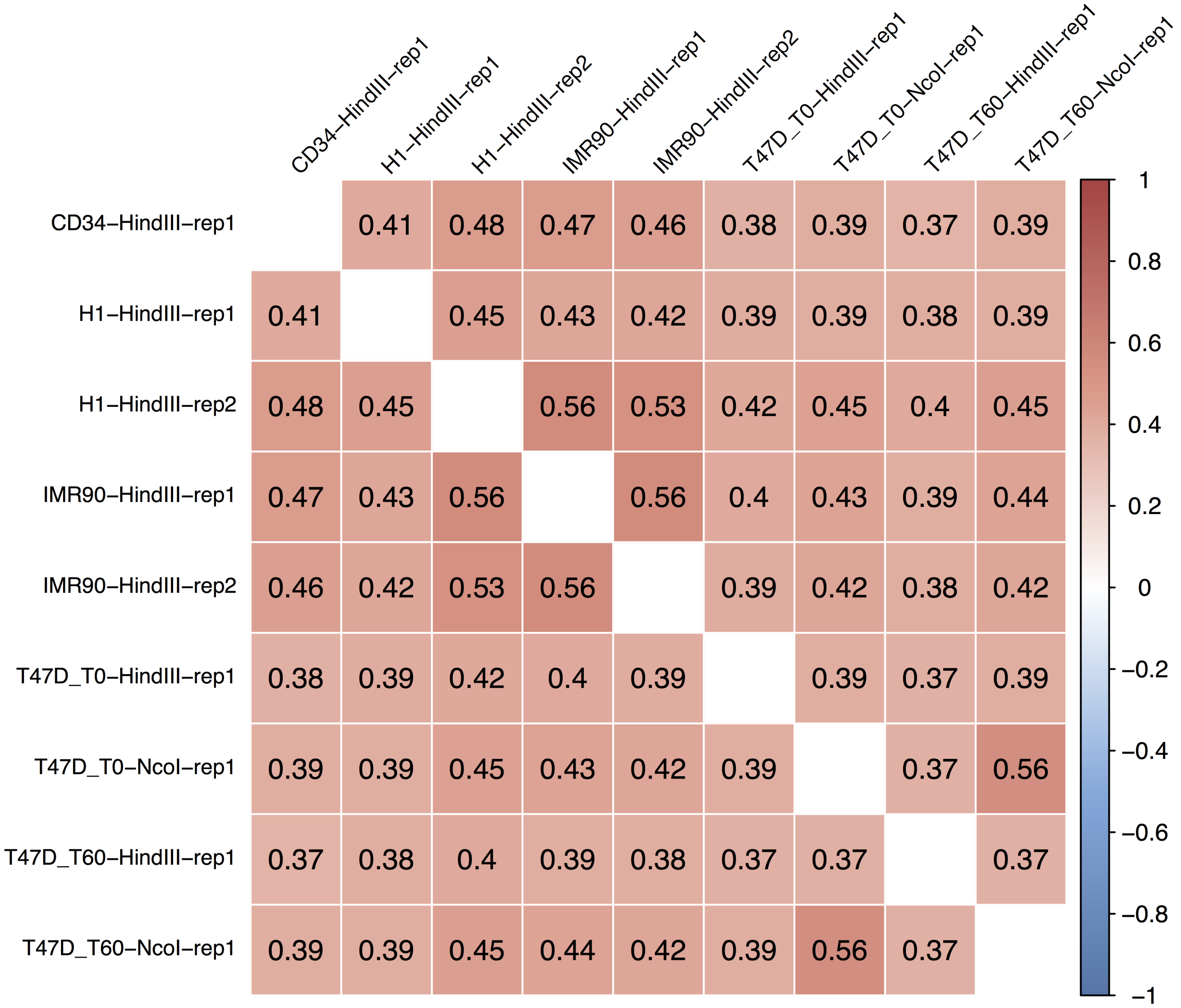

Figure 7: Compare Matrices Stats Spearman sample correlograms. See Section 4.12.

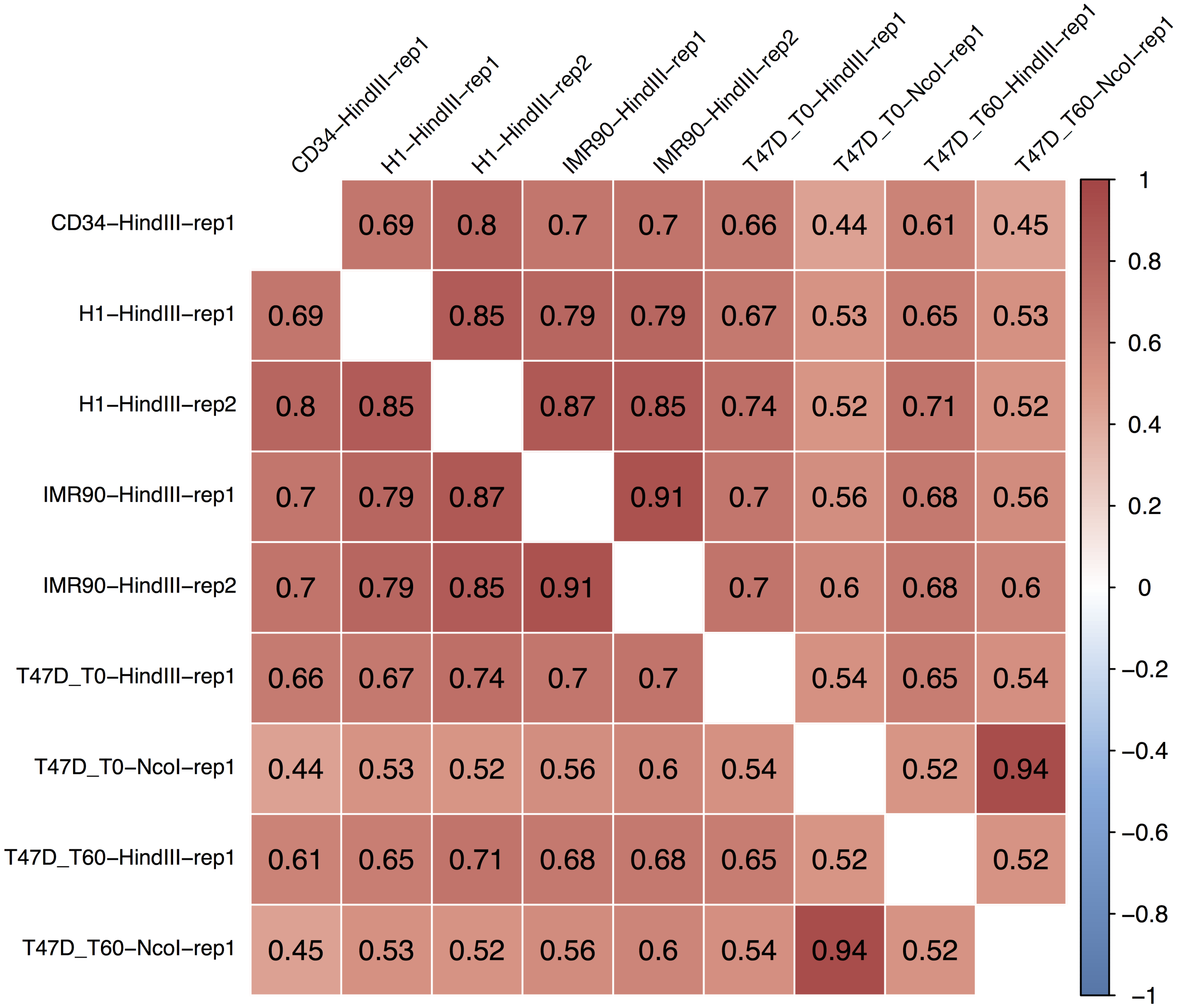

Figure 8: Compare Matrices Stats Pearson sample correlograms. See Section 4.12.

4.13 Boundary Scores

4.13.1 Input

Data from the pipeline steps `matrix-filtered` (Section 4.6), `matrix-hicnorm` (Section 4.9), `matrix-prep` (Section 4.7), and `matrix-ic` (Section 4.8) are used as input.

4.13.2 Analysis

Default parameters:

```
params.standard.tcsh$
#!/bin/tcsh

source ./inputs/params/params.tcsh

set chrom_excluded = 'chr[MYX]'                                        # excluded chromosomes

set boundary_scores_params = ( \
--min-lambda=0.0 --max-lambda=1.0 --n-lambda=6 --gamma=0 \
--preprocess=none \
--distance=`echo 500000/$bin_size | bc` \
--distance2=`echo 500000/$bin_size | bc` \
--skip-distance=0 \
--flank-dist=`echo 500000/$bin_size | bc` \
--tolerance=0.01 \
--alpha=0.50 \
--track-dist=`echo 2000000/$bin_size | bc` \
--presentation=none \
)
```

4.13.3 Output

Default output:

```
-rw-r--r--  1 at570  9.0M Feb 15 14:25 all_scores.k=001.tsv
-rw-r--r--  1 at570   17K Feb 15 14:25 job.err
-rw-r--r--  1 at570    47 Feb 15 14:07 job.id
-rw-r--r--  1 at570     0 Feb 15 14:11 job.out
-rw-r--r--  1 at570   345 Feb 15 14:07 job.sh
-rw-r--r--  1 at570  3.2K Feb 15 14:25 job.vars.tsv
```

4.14 Boundary Scores PCA

4.14.1 Input

Data from the pipeline `boundary-scores` step is used as input (Section 4.13).

4.14.2 Analysis

Default parameters:

```
params.standard.tcsh
#!/bin/tcsh

source ./inputs/params/params.tcsh

set chrom_excluded = 'chr[MYX]'              # excluded chromosomes

set group_var = 'cell_type'                  # grouping variable (from sample sheet) to be used for color
    assignment)
```

4.14.3 Output

See Figure 9. Default output:

```
-rw-r--r-- 1 at570 4.1K Feb 15 15:20 job.err
-rw-r--r-- 1 at570   47 Feb 15 15:18 job.id
-rw-r--r-- 1 at570  936 Feb 15 15:20 job.out
-rw-r--r-- 1 at570  564 Feb 15 15:18 job.sh
-rw-r--r-- 1 at570 4.8K Feb 15 15:20 job.vars.tsv
-rw-r--r-- 1 at570  211 Feb 15 15:18 labels.tsv
-rw-r--r-- 1 at570 4.4K Feb 15 15:19 pca.DI.k=001.pdf
-rw-r--r-- 1 at570 4.4K Feb 15 15:19 pca.DI2.k=001.pdf
-rw-r--r-- 1 at570 4.4K Feb 15 15:19 pca.diff.k=001.pdf
-rw-r--r-- 1 at570 4.4K Feb 15 15:20 pca.diffratio.k=001.pdf
-rw-r--r-- 1 at570 4.4K Feb 15 15:19 pca.inter.k=001.pdf
-rw-r--r-- 1 at570 4.4K Feb 15 15:18 pca.intra-left.k=001.pdf
-rw-r--r-- 1 at570 4.4K Feb 15 15:19 pca.intra-max.k=001.pdf
-rw-r--r-- 1 at570 4.4K Feb 15 15:19 pca.intra-min.k=001.pdf
-rw-r--r-- 1 at570 4.4K Feb 15 15:18 pca.intra-right.k=001.pdf
-rw-r--r-- 1 at570 4.4K Feb 15 15:20 pca.novel-max.k=001.pdf
-rw-r--r-- 1 at570 4.4K Feb 15 15:20 pca.novel-min.k=001.pdf
-rw-r--r-- 1 at570 4.4K Feb 15 15:19 pca.ratio.k=001.pdf
```

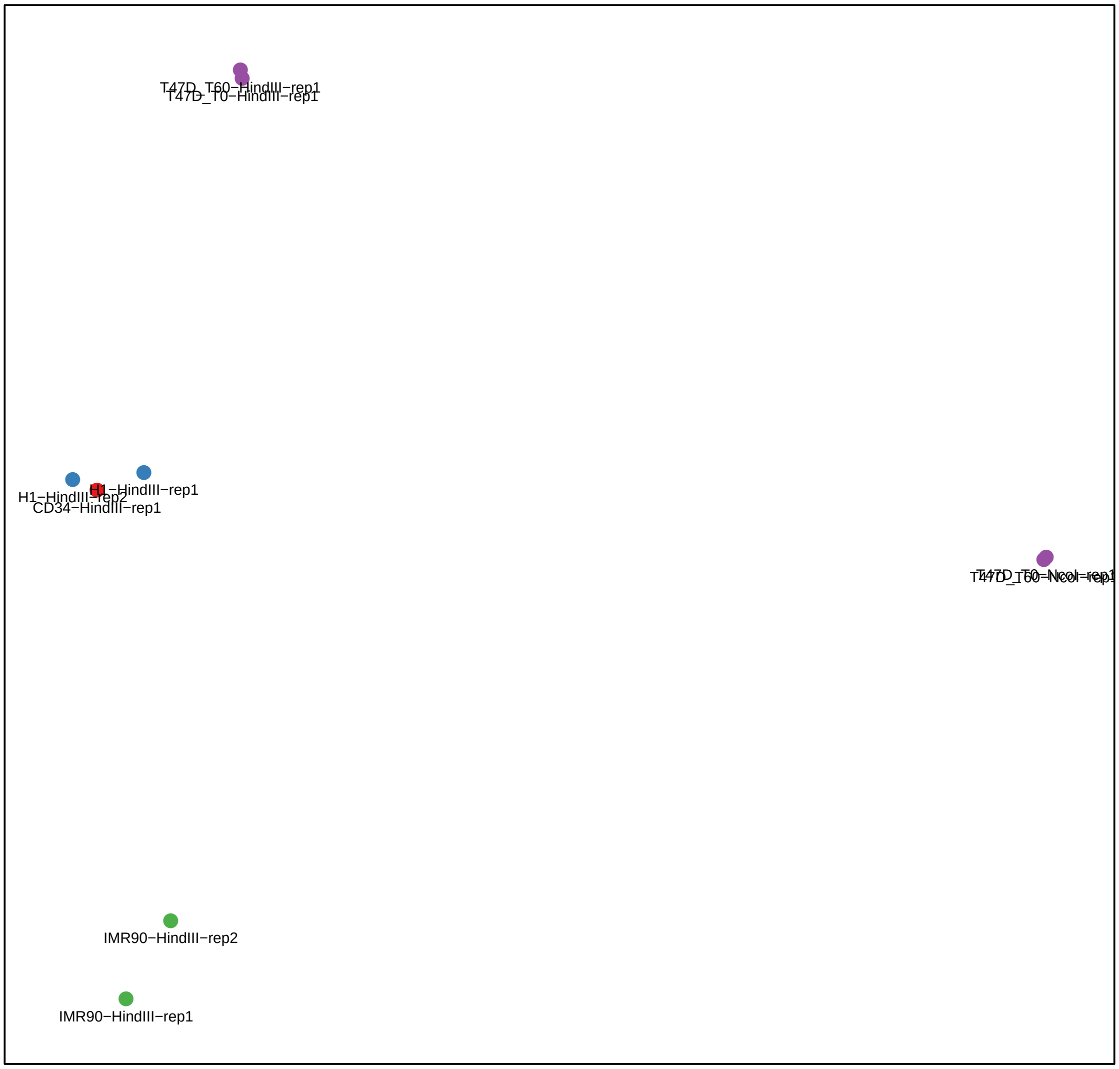

Figure 9: Boundary Scores PCA sample output. See Section 4.14.

4.15 Domains

4.15.1 Input

Data from the pipeline steps `matrix-filtered` (Section 4.6), `matrix-hicnorm` (Section 4.9), `matrix-prep` (Section 4.7), and `matrix-ic` (Section 4.8) are used as input.

4.15.2 Analysis

Default parameters:

```
params.armatus.gamma_0.5.tcsh$
#!/bin/tcsh

source ./inputs/params/params.tcsh

set tool = armatus
set chrom_excluded = 'chr[MY]'
set armatus_params = "-g 0.5"
```

```
params.hicmatrix.tcsh$
#!/bin/tcsh

source ./inputs/params/params.tcsh

set tool = hicmatrix

set chrom_excluded = 'chr[MY]'

set hicmatrix_params = ( \
--min-lambda=0.0 --max-lambda=1.0 --n-lambda=6 --gamma=0 \
--preprocess=none \
--method=ratio \
--distance=`echo 500000/$bin_size | bc` \
--distance2=`echo 500000/$bin_size | bc` \
--skip-distance=0 \
--flank-dist=`echo 500000/$bin_size | bc` \
--tolerance=0.01 \
--alpha=0.25 \
--track-dist=`echo 2000000/$bin_size | bc` \
--presentation=none \
)
```

```
params.topdom.tcsh$
#!/usr/bin/tcsh

source ./inputs/params/params.tcsh

set tool = topdom
set topdompath = "./code/TopDom.R"
set chrom_excluded = 'chr[MY]'
set winsize = 5
```

4.15.3 Output

Default output:

```
-rw-r--r-- 1 at570 288K Feb 15 16:31 domains.k=001.bed
-rw-r--r-- 1 at570  28K Feb 15 16:31 job.err
-rw-r--r-- 1 at570   47 Feb 15 16:13 job.id
-rw-r--r-- 1 at570 5.6K Feb 15 16:31 job.out
-rw-r--r-- 1 at570  347 Feb 15 16:13 job.sh
-rw-r--r-- 1 at570 2.7K Feb 15 16:31 job.vars.tsv
```

4.16 Compare Boundaries

4.16.1 Input

Data from the pipeline `domains` step is used as input (Section 4.15).

4.16.2 Analysis

Default parameters:

```
params.standard.tcsh$
#!/bin/tcsh

source ./inputs/params/params.tcsh

set flank_dist = $bin_size
set black_lists = ($genome_dir/centrotelo.bed)
```

4.16.3 Output

Default output:

```
-rw-r--r--  1 at570 573K Feb 16 00:18 boundaries1.k=001.bed
-rw-r--r--  1 at570 573K Feb 16 00:18 boundaries2.k=001.bed
-rw-r--r--  1 at570 268K Feb 16 00:18 common_boundaries.k=001.bed
-rw-r--r--  1 at570  154 Feb 16 00:18 comparison.tsv
-rw-r--r--  1 at570 268K Feb 16 00:18 intersection.k=001.bed
-rw-r--r--  1 at570   70 Feb 16 00:18 job.err
-rw-r--r--  1 at570   47 Feb 16 00:18 job.id
-rw-r--r--  1 at570    0 Feb 16 00:18 job.out
-rw-r--r--  1 at570  456 Feb 16 00:18 job.sh
-rw-r--r--  1 at570 4.5K Feb 16 00:18 job.vars.tsv
-rw-r--r--  1 at570 268K Feb 16 00:18 union.k=001.bed
```

4.17 Compare Boundaries Stats

4.17.1 Input

Data from the pipeline `compare-boundaries` step is used as input (Section 4.16).

4.17.2 Analysis

Default parameters:

```
params.standard.tcsh$
#!/bin/tcsh

source ./inputs/params/params.tcsh
```

4.17.3 Output

See Figure 11 and Figure 10. Default output:

```
-rw-r--r-- 1 at570 6.9K Feb 12 12:52 comparisons.tsv
-rw-r--r-- 1 at570  27K Feb 12 12:52 correlograms.pdf
-rw-r--r-- 1 at570  238 Feb 12 12:52 job.err
-rw-r--r-- 1 at570   47 Feb 12 12:51 job.id
-rw-r--r-- 1 at570   52 Feb 12 12:52 job.out
-rw-r--r-- 1 at570 3.5K Feb 12 12:51 job.sh
-rw-r--r-- 1 at570  51K Feb 12 12:52 job.vars.tsv
-rw-r--r-- 1 at570 5.6K Feb 12 12:52 raw_comparisons.pdf
```

Number of boundaries

	CD34–HindIII–rep1	H1–HindIII–rep1	H1–HindIII–rep2	IMR90–HindIII–rep1	IMR90–HindIII–rep2	T47D_T0–HindIII–rep1	T47D_T0–NcoI–rep1	T47D_T60–HindIII–rep1	T47D_T60–NcoI–rep1
CD34–HindIII–rep1	**9158**	**5231**	**5277**	**4045**	**4157**	**5006**	**3532**	**4869**	**3546**
H1–HindIII–rep1	**5231**	**7258**	**4724**	**3860**	**4059**	**4481**	**3215**	**4481**	**3232**
H1–HindIII–rep2	**5277**	**4724**	**6564**	**3615**	**3719**	**4186**	**2860**	**4149**	**2890**
IMR90–HindIII–rep1	**4045**	**3860**	**3615**	**5464**	**4291**	**3539**	**2666**	**3576**	**2678**
IMR90–HindIII–rep2	**4157**	**4059**	**3719**	**4291**	**5800**	**3767**	**2813**	**3749**	**2823**
T47D_T0–HindIII–rep1	**5006**	**4481**	**4186**	**3539**	**3767**	**7364**	**3252**	**5027**	**3264**
T47D_T0–NcoI–rep1	**3532**	**3215**	**2860**	**2666**	**2813**	**3252**	**5417**	**3205**	**4467**
T47D_T60–HindIII–rep1	**4869**	**4481**	**4149**	**3576**	**3749**	**5027**	**3205**	**7215**	**3234**
T47D_T60–NcoI–rep1	**3546**	**3232**	**2890**	**2678**	**2823**	**3264**	**4467**	**3234**	**5401**

Figure 10: Example raw comparisons. See Section 4.17.

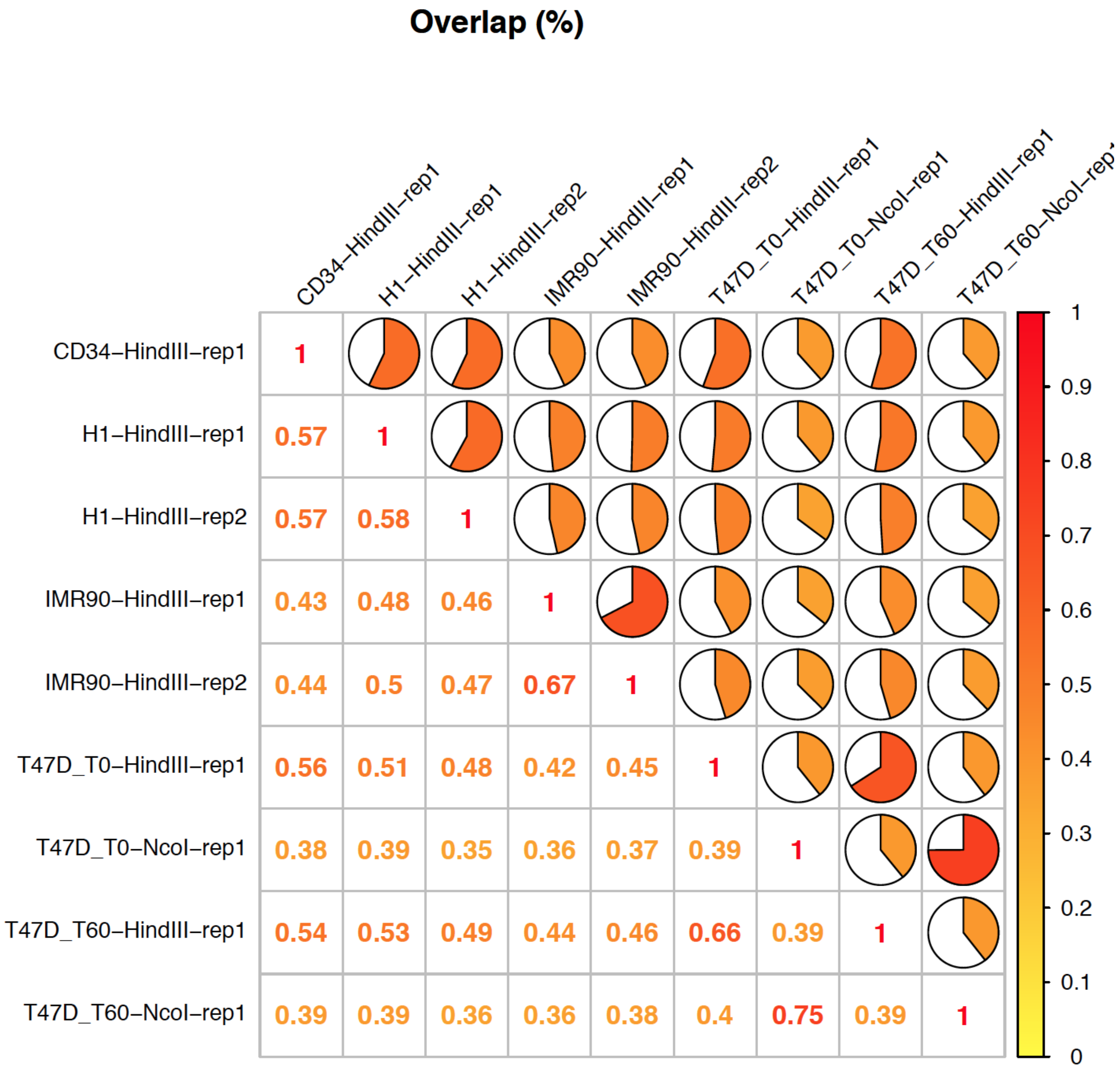

Figure 11: Example correlograms. See Section 4.17.

4.18 HiC Plotter

4.18.1 Input

Data from the pipeline steps `matrix-filtered` (Section 4.6), `matrix-hicnorm` (Section 4.9), `matrix-prep` (Section 4.7), and `matrix-ic` (Section 4.8) are used as input.

4.18.2 Analysis

Default parameters:

```
params.standard.tcsh$
#!/bin/tcsh

source ./inputs/params/params.tcsh

# HiCplotter path
set hicplotter_path = ./code/HiCPlotter2.py

# create bedgraphs for boundary scores
set bscores_branch = ../boundary-scores/results/boundary-scores.by_sample.standard/`echo $branch | sed 's/.*
    results\///''`
set cell_type = `echo $objects[1] | cut -d'-' -f1`
set f = $bscores_branch/$objects[1]/all_scores.k=001.tsv
set methods = (intra-max DI ratio)
set bedgraphs = ()
set bedgraph_labels = ($methods)
foreach m ($methods)
  set k = `head -1 $f | tr '\t' '\n' | grep -n "^$m"'$' | cut -d':' -f1`
  cat $f | sed '1d' | cut -f1,$k | sed 's/:/\t/' | sed 's/-/\t/' >! $outdir/bscores.$m.bedGraph
  set bedgraphs = ($bedgraphs $outdir/bscores.$m.bedGraph)
end

# add CTCF ChIP-seq
if (-e inputs/data.external/$cell_type/CTCF.bedGraph) then
  set bedgraphs = ($bedgraphs inputs/data.external/$cell_type/CTCF.bedGraph)
  set bedgraph_labels = ($bedgraph_labels CTCF)
endif

# regions to plot
set regions = "chr8:125000000-133000000"
set tiles = "params/regions.bed"
set tiles_labels = "regions"
set highlight = 1
set highlight_bed = "params/highlight.bed"
set fileheader = 0          # Either 1 or 0 (header / no header)
set insulation_score = 0    # Either 1 or 0 (include insulation index or not)
```

4.18.3 Output

See Figure 12. Default output:

```
-rw-r--r--  1 at570  2.3M Feb 15 14:49 bscores.DI.bedGraph
-rw-r--r--  1 at570  2.3M Feb 15 14:49 bscores.intra-max.bedGraph
-rw-r--r--  1 at570  2.3M Feb 15 14:49 bscores.ratio.bedGraph
-rw-r--r--  1 at570  146K Feb 15 14:50 chr8:125000000-133000000.pdf
-rw-r--r--  1 at570   107 Feb 15 14:50 job.err
-rw-r--r--  1 at570    47 Feb 15 14:49 job.id
-rw-r--r--  1 at570    40 Feb 15 14:50 job.out
-rw-r--r--  1 at570   335 Feb 15 14:49 job.sh
-rw-r--r--  1 at570  8.4K Feb 15 14:50 job.vars.tsv
```

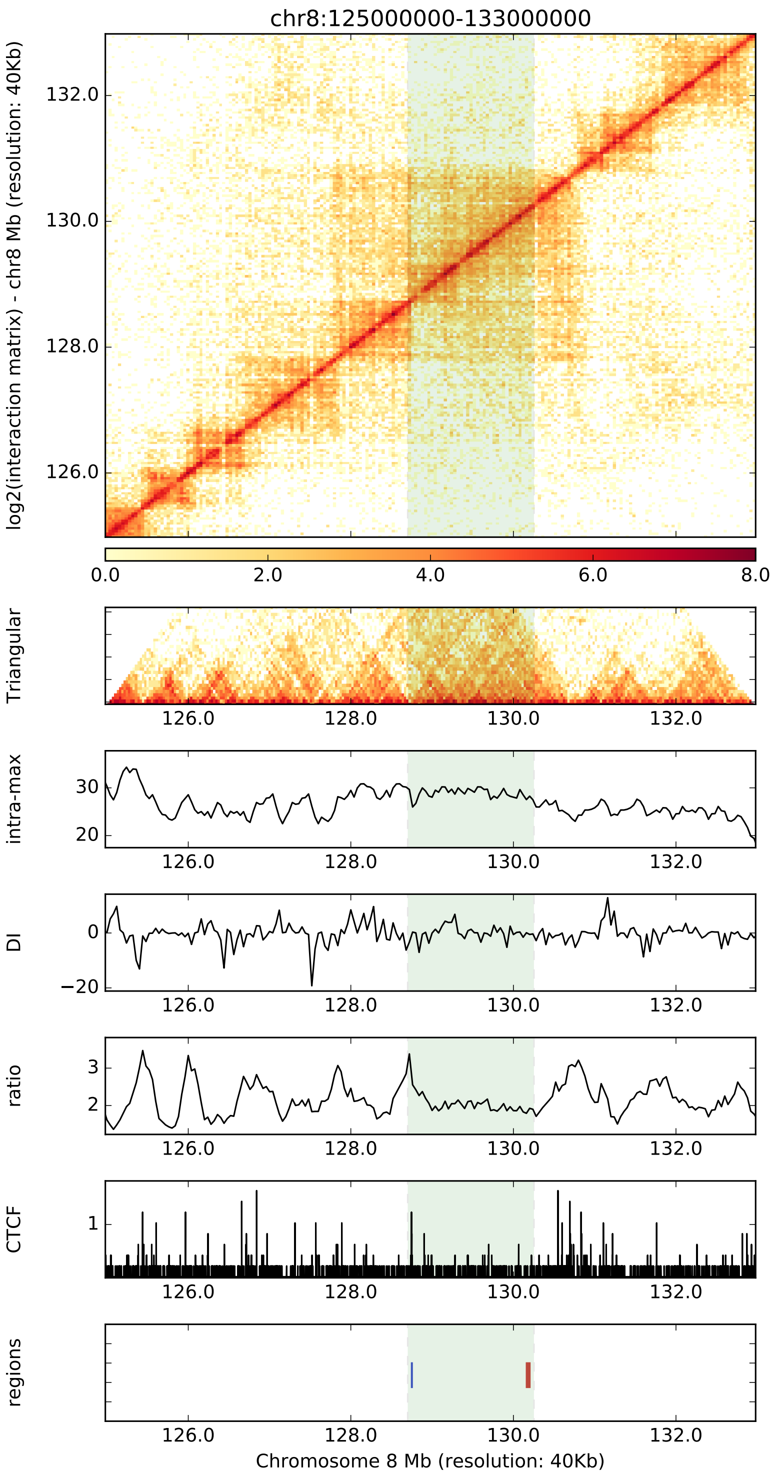

Figure 12: HiCPlotter sample output

4.19 Interactions

4.19.1 Input

Data from the pipeline `matrix-filtered` step is used as input (Section 4.6).

4.19.2 Analysis

Default parameters:

```
#!/bin/tcsh

source ./inputs/params/params.tcsh

set chrom_excluded = 'chr[MYX]'       # excluded chromosomes

set loop_params = "--bin-size=$bin_size --lambda-id=6 --rpk2b-cutoff=1.0 --loop-cutoff=4.0 --min-distance=40000"           # parameters for identifying significant interactions
```

4.19.3 Output

See Figure 13. Default output:

```
drwxr-xr-x  2 at570 3.3K Feb  5 10:12  __jdata
-rw-r--r--  1 at570 4.8K Feb  5 10:17  job.err
-rw-r--r--  1 at570   47 Feb  5 10:11  job.id
-rw-r--r--  1 at570    0 Feb  5 10:11  job.out
-rw-r--r--  1 at570  375 Feb  5 10:11  job.sh
-rw-r--r--  1 at570 3.6K Feb  5 10:17  job.vars.tsv
drwxr-xr-x  2 at570   54 Feb  5 10:15  matrix.chr1
drwxr-xr-x  2 at570   54 Feb  5 10:14  matrix.chr10
drwxr-xr-x  2 at570   54 Feb  5 10:14  matrix.chr11
drwxr-xr-x  2 at570   54 Feb  5 10:14  matrix.chr12
drwxr-xr-x  2 at570   54 Feb  5 10:13  matrix.chr13
drwxr-xr-x  2 at570   54 Feb  5 10:13  matrix.chr14
drwxr-xr-x  2 at570   54 Feb  5 10:13  matrix.chr15
drwxr-xr-x  2 at570   54 Feb  5 10:13  matrix.chr16
drwxr-xr-x  2 at570   54 Feb  5 10:13  matrix.chr17
drwxr-xr-x  2 at570   54 Feb  5 10:13  matrix.chr18
drwxr-xr-x  2 at570   54 Feb  5 10:13  matrix.chr19
drwxr-xr-x  2 at570   54 Feb  5 10:16  matrix.chr2
drwxr-xr-x  2 at570   54 Feb  5 10:13  matrix.chr20
drwxr-xr-x  2 at570   54 Feb  5 10:12  matrix.chr21
drwxr-xr-x  2 at570   54 Feb  5 10:13  matrix.chr22
drwxr-xr-x  2 at570   54 Feb  5 10:15  matrix.chr3
drwxr-xr-x  2 at570   54 Feb  5 10:15  matrix.chr4
drwxr-xr-x  2 at570   54 Feb  5 10:15  matrix.chr5
drwxr-xr-x  2 at570   54 Feb  5 10:15  matrix.chr6
drwxr-xr-x  2 at570   54 Feb  5 10:14  matrix.chr7
drwxr-xr-x  2 at570   54 Feb  5 10:14  matrix.chr8
drwxr-xr-x  2 at570   54 Feb  5 10:14  matrix.chr9
```

```
matrix.chr1$
-rw-r--r--  1 at570 4.7M Feb  5 10:15  loops.tsv
-rw-r--r--  1 at570  27K Feb  5 10:16  plots.pdf
```

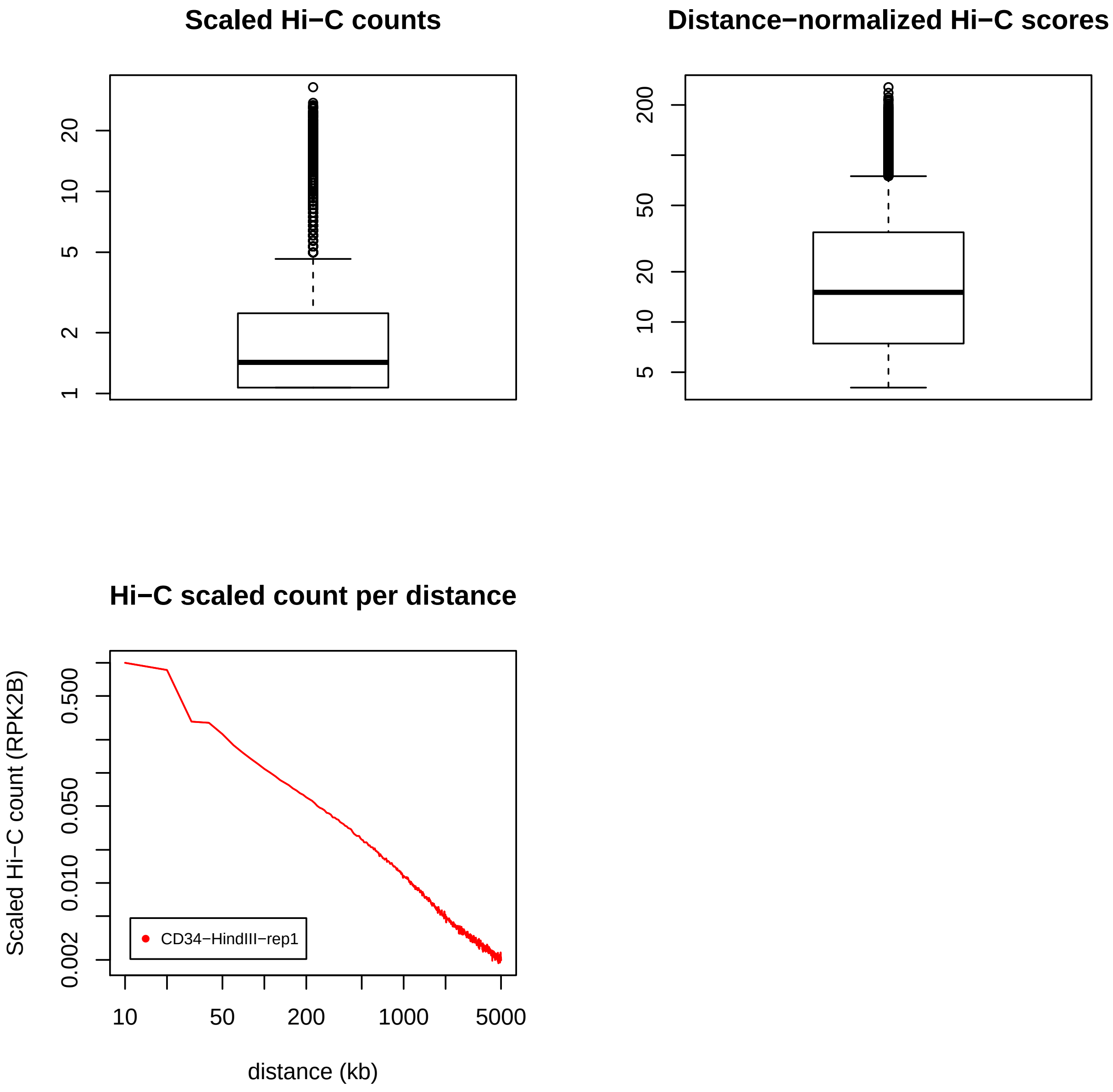

Figure 13: Interactions sample output

4.20 Annotations

4.20.1 Input

Data from the pipeline `interactions` step is used as input (Section 4.19).

4.20.2 Analysis

Default parameters:

```
params.standard.tcsh$
#!/bin/tcsh

source ./inputs/params/params.tcsh

set genes_bed = $genome_dir/gene.bed                          # gene BED6 file for annotation of interactions
set cell_type = `echo $objects[1] | cut -d'-' -f1`
if (! -e inputs/data.external/$cell_type) then
  set loci_bed = ()
else
  set loci_bed = `find inputs/data.external/$cell_type -maxdepth 1 -name '*.bed'`
endif
```

4.20.3 Output

Default output:

```
-rw-r--r--  1 at570  5.9M Feb  5 17:33 bin.annotated.tsv
-rw-r--r--  1 at570  3.8M Feb  5 17:33 bin.gene.tsv
-rw-r--r--  1 at570  7.5M Feb  5 17:33 bin.loci.tsv
-rw-r--r--  1 at570  8.7M Feb  5 17:33 bin.reg
-rw-r--r--  1 at570  5.4K Feb  5 17:33 job.err
-rw-r--r--  1 at570    47 Feb  5 17:32 job.id
-rw-r--r--  1 at570     0 Feb  5 17:33 job.out
-rw-r--r--  1 at570   434 Feb  5 17:32 job.sh
-rw-r--r--  1 at570  3.1K Feb  5 17:33 job.vars.tsv
-rw-r--r--  1 at570   42M Feb  5 17:33 loci.reg
-rw-r--r--  1 at570   45M Feb  5 17:33 table.annotated.tsv
```

4.21 Annotations Stats

4.21.1 Input

Data from the pipeline `annotations` step is used as input (Section 4.20).

4.21.2 Analysis

Default parameters:

```
params.standard.tcsh$
#!/bin/tcsh

source ./inputs/params/params.tcsh

set nbest = 10000          # choose top-scoring interactions to calculate enrichments
```

4.21.3 Output

See Figure 14. Default output:

```
-rw-r--r--  1 at570   77 Feb 16 17:26 counts.tsv
-rw-r--r--  1 at570  350 Feb 16 17:26 enrich.tsv
-rw-r--r--  1 at570 7.0K Feb 16 17:26 enrichment.pdf
-rw-r--r--  1 at570  121 Feb 16 17:26 job.err
-rw-r--r--  1 at570   47 Feb 16 17:25 job.id
-rw-r--r--  1 at570   62 Feb 16 17:26 job.out
-rw-r--r--  1 at570  507 Feb 16 17:25 job.sh
-rw-r--r--  1 at570 3.1K Feb 16 17:26 job.vars.tsv
-rw-r--r--  1 at570  184 Feb 16 17:25 top_counts.tsv
```

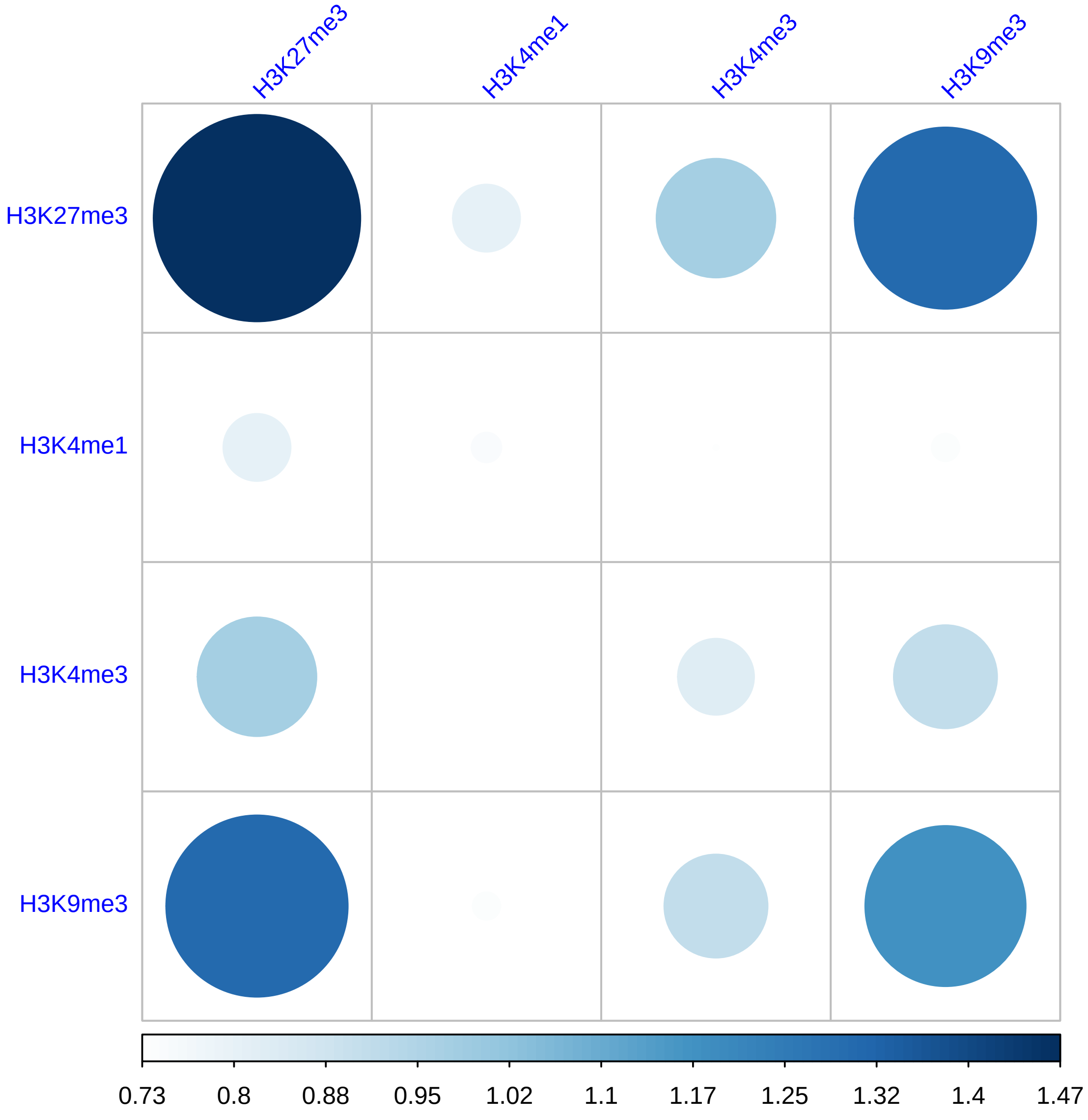

Figure 14: Annotation Stats enrichment sample output. See Section 4.21.

5 Appendix

5.1 Error Logs

Errors encountered during pipeline execution can be viewed with:

```
<project_directory>$ code.main/pipeline-errors
```

Analysis results can be removed with:

```
<project_directory>$ code/clean-all
```

5.2 Other Pipeline Software: gtools-hic

```
code.repo/bin/gtools-hic$

USAGE:
  gtools-hic OPERATION [OPTIONS] <REGION-SET>

VERSION:
  genomic-tools 3.0.0

DESCRIPTION:
  Pipeline for HiC-seq data analysis. For detailed description and list of options choose an operation and use
    the --help option.

OPERATION:
  align          Iteratively aligns HiC-seq read pairs to reference genome using bowtie2.
  classify       Classifies and computes various metrics for HiC-seq aligned read pairs.
  filter         Filters HiC-seq aligned read pairs for common experimental artifacts.
  bin            Bins filtered read pairs to genomic bins of desired resolution.
  matrix         Create Hi-C count matrix.
  convert        Convert contact matrix into WashU Epigenome Browser format.
```

5.2.1 gtools-hic align

```
code.repo/bin/gtools-hic align --help

USAGE:
  gtools-hic align [OPTIONS] READ1-FASTQ READ2-FASTQ

DESCRIPTION:
  Iteratively aligns HiC-seq read pairs to reference genome using bowtie2.

DETAILS:
  * Input: FASTQ files
  * Output: aligned reads in SAM format (same order as in fastq files)

OPTIONS:
  --help                    help                                          [true]
  -h                        help                                          [true]
  -v                        verbose mode                                  [false]
  --work-dir                working directory (required)                  []
  --min-len                 minimum truncated read length                 [30]
  --len-diff                read truncation step                          [10]
  -p                        number of threads for bowtie2 run             [1]
  --bowtie-path             full bowtie2 path (version>=2.1.0)            [bowtie2]
  --bowtie-index            full bowtie2 index prefix path                [genome/bowtie2.index
    /genome]
```

5.2.2 gtools-hic classify

```
code.repo/bin/gtools-hic classify --help$

USAGE:
  gtools-hic classify [OPTIONS] <ALIGNED-READS>

DESCRIPTION:
  Classifies and computes various metrics for HiC-seq aligned read pairs.

DETAILS:
  * Input: aligned reads in SAM format (sorted by read-id, at most one alignment per read)
  * Output: tab-separated table

OPTIONS:
  --help                   help                                                  [true]
  -h                       help                                                  [true]
  -v                       verbose mode                                          [false]
  -E                       enzyme fragments (BED/GFF/SAM/REG)                    []
  --mapq                   minimum mapping quality (MAPQ)                        [3.000000e+01]
  --min-dist               miminum allowed distance between 5's of reads in read pair     [500]
  --max-offset             maximum allowed offset of 5's of reads from fragment ends      [500]
```

5.2.3 gtools-hic filter

```
code.repo/bin/gtools-hic filter --help$

USAGE:
  gtools-hic filter [OPTIONS] <ALIGNED-READS>

DESCRIPTION:
  Filters HiC-seq aligned read pairs for common experimental artifacts.

DETAILS:
  * Input: aligned reads in SAM format (sorted by read-id, at most one alignment per read)
  * Output: filtered read pairs in REG format

OPTIONS:
  --help                   help                                                  [true]
  -h                       help                                                  [true]
  -v                       verbose mode                                          [false]
  -E                       enzyme fragments (BED/GFF/SAM/REG)                    []
  --mapq                   minimum mapping quality (MAPQ)                        [3.000000e+01]
  --min-dist               miminum allowed distance between 5's of reads in read pair     [500]
  --max-offset             maximum allowed offset of 5's of reads from fragment ends      [500]
  --filter-dups            filter duplicate read pairs as PCR artifacts          [false]
  --stats                  output statistics file (default=stderr)               []
```

5.2.4 gtools-hic bin

```
code.repo/bin/gtools-hic bin --help$

USAGE:
  gtools-hic bin [OPTIONS] <FILTERED-READ-PAIRS>

DESCRIPTION:
  Bins filtered read pairs to genomic bins of desired resolution.

DETAILS:
  * Input: filtered read pairs in REG format
  * Output: binned read pairs

OPTIONS:
  --help                   help                                                  [true]
  -h                       help                                                  [true]
  -v                       verbose mode                                          [false]
  --bin-size               genomic bin size                                      [1000000]
  -g                       genome region file (BED/REG)                          []
```

```
  --split-matrix             print output as matrix                                   [false]
  --matrix                   print output as matrix (overrides --split-matrix)        [false]
```

5.2.5 gtools-hic matrix

```
code.repo/bin/gtools-hic matrix --help$

USAGE:
  gtools-hic matrix [OPTIONS] <FILTERED-READ-PAIRS>

DESCRIPTION:
  Create Hi-C count matrix.

DETAILS:
  * Input: filtered read pairs in REG format
  * Output: contact matrix

OPTIONS:
  --help                     help                                                     [true]
  -h                         help                                                     [true]
  -v                         verbose mode                                             [false]
  --bin-size                 genomic bin size (in nucleotides)                        [5000]
  --max-dist                 maximum distance between bins (in nucleotides; default = no restriction)[0]
  --rotate45                 rotate matrix by 45 degrees (applicable if --max-dist > 0)     [false]
  -R                         reference region file (BED/REG)                          []
  -p                         output file prefix                                       []
```

5.2.6 gtools-hic convert

```
code.repo/bin/gtools-hic convert --help

USAGE:
  gtools-hic convert [OPTIONS] <CONTACT-MATRIX>

DESCRIPTION:
  Convert contact matrix into WashU Epigenome Browser format.

DETAILS:
  * Input: locus-labelled contact matrix
  * Output: WashU Epigenome Browser format

OPTIONS:
  --help                     help                                                     [true]
  -h                         help                                                     [true]
  -v                         verbose mode                                             [false]
  --col-labels               input matrix has column labels                           [false]
  -t                         matrix element separator                                 [ ]
  -c                         normalization constant                                   [1.000000e+00]
  -min                       score cutoff (values below this are set to zero)         [0.000000e+00]
  -d                         maximum distance between interacting loci (default = no limit) [0]
```

5.3 Other Pipeline Software: pipeline-master-explorer.r

The `pipeline-master-explorer.r` script, located in the `code.main` directory, is the driver of combinatorial parameter exploration during the execution of each pipeline step.

```
code.main$ ./pipeline-master-explorer.r --help
Usage: pipeline-master-explorer.r [OPTIONS] SCRIPT OUTDIR-PREFIX PARAM-SCRIPTS INPUT-BRANCHES SPLIT-VARIABLE
     OUTPUT-OBJECT-VARIABLE TUPLES


Options:
  -v, --verbose
     Print more messages.

  -S SAMPLE-SHEET, --sample-sheet=SAMPLE-SHEET
     Sample sheet file name (required) [default "inputs/sample-sheet.tsv"].

  -F FILTER-BRANCH, --filter-branch=FILTER-BRANCH
     Regular expression for filtering input branches [default ""].

  --exclude-branch=EXCLUDE-BRANCH
     Regular expression for excluding input branches [default ""].

  --exclude-obj=EXCLUDE-OBJ
     Regular expression for excluding input objects [default ""].

  --exclude-outdir=EXCLUDE-OUTDIR
     Regular expression for excluding output directories [default ""].

  -h, --help
     Show this help message and exit
```

5.4 System and Session Information

LaTeX version: LaTeX 2_ε 2005/12/01

```r
system('uname -srv',intern=T)

## [1] "Linux 2.6.32-573.18.1.el6.x86_64 #1 SMP Tue Feb 9 22:46:17 UTC 2016"

sessionInfo()

## R version 3.2.3 (2015-12-10)
## Platform: x86_64-redhat-linux-gnu (64-bit)
## Running under: CentOS release 6.7 (Final)
##
## locale:
##  [1] LC_CTYPE=en_US.UTF-8       LC_NUMERIC=C               LC_TIME=en_US.UTF-8
##  [4] LC_COLLATE=en_US.UTF-8     LC_MONETARY=en_US.UTF-8    LC_MESSAGES=en_US.UTF-8
##  [7] LC_PAPER=en_US.UTF-8       LC_NAME=C                  LC_ADDRESS=C
## [10] LC_TELEPHONE=C             LC_MEASUREMENT=en_US.UTF-8 LC_IDENTIFICATION=C
##
## attached base packages:
## [1] stats     graphics  grDevices utils     datasets  methods   base
##
## other attached packages:
## [1] knitr_1.12.3
##
## loaded via a namespace (and not attached):
## [1] magrittr_1.5   formatR_1.2.1 tools_3.2.3   stringi_1.0-1 highr_0.5.1   stringr_1.0.0
## [7] evaluate_0.8
```

5.4.1 LaTeX File List

```
 article.cls    2005/09/16 v1.4f Standard LaTeX document class
  size10.clo    2005/09/16 v1.4f Standard LaTeX file (size option)
graphicx.sty    1999/02/16 v1.0f Enhanced LaTeX Graphics (DPC,SPQR)
  keyval.sty    1999/03/16 v1.13 key=value parser (DPC)
graphics.sty    2006/02/20 v1.0o Standard LaTeX Graphics (DPC,SPQR)
    trig.sty    1999/03/16 v1.09 sin cos tan (DPC)
graphics.cfg    2007/01/18 v1.5 graphics configuration of teTeX/TeXLive
  pdftex.def    2007/01/08 v0.04d Graphics/color for pdfTeX
   color.sty    1999/02/16
   color.cfg    2007/01/18 v1.5 color configuration of teTeX/TeXLive
  framed.sty    2011/10/22 v 0.96: framed or shaded text with page breaks
   alltt.sty    1997/06/16 v2.0g defines alltt environment
mathpazo.sty    2005/04/12 PSNFSS-v9.2a Palatino w/ Pazo Math (D.Puga, WaS)
  helvet.sty    2005/04/12 PSNFSS-v9.2a (WaS)
 fontenc.sty
   t1enc.def    2005/09/27 v1.99g Standard LaTeX file
geometry.sty    2002/07/08 v3.2 Page Geometry
geometry.cfg
    cite.sty    2003/11/04  v 4.01
 caption.sty    2007/01/07 v3.0k Customising captions (AR)
caption3.sty    2007/01/07 v3.0k caption3 kernel (AR)
hyperref.sty    2007/02/07 v6.75r Hypertext links for LaTeX
  pd1enc.def    2007/02/07 v6.75r Hyperref: PDFDocEncoding definition (HO)
hyperref.cfg    2002/06/06 v1.2 hyperref configuration of TeXLive
kvoptions.sty    2006/08/22 v2.4 Connects package keyval with LaTeX options (HO
)
   puenc.def    2007/02/07 v6.75r Hyperref: PDF Unicode definition (HO)
     url.sty    2005/06/27  ver 3.2  Verb mode for urls, etc.
 hpdftex.def    2007/02/07 v6.75r Hyperref driver for pdfTeX
breakurl.sty    2006/08/26 v1.20 Breakable hyperref URLs
 xkeyval.sty    2006/11/18 v2.5f package option processing (HA)
 xkeyval.tex    2006/11/18 v2.5f key=value parser (HA)
```

```
 forloop.sty    2006/09/18 v3.0 For Loops for LaTeX
  ifthen.sty    2001/05/26 v1.1c Standard LaTeX ifthen package (DPC)
    tikz.sty    2006/10/17 v1.10 (rcs-revision 1.68)
     pgf.sty    2006/10/11 v1.10 (rcs-revision 1.7)
  pgfrcs.sty    2006/10/26 v1.10 (rcs-revision 1.14)
  pgfrcs.code.tex
 pgfcore.sty    2006/10/11 v1.10 (rcs-revision 1.4)
  pgfsys.sty    2006/10/16 v1.10 (rcs-revision 1.19)
  pgfsys.code.tex
pgfsyssoftpath.code.tex    2006/10/16  (rcs-revision 1.4)
pgfsysprotocol.code.tex    2006/10/16  (rcs-revision 1.4)
  xcolor.sty    2007/01/21 v2.11 LaTeX color extensions (UK)
   color.cfg    2007/01/18 v1.5 color configuration of teTeX/TeXLive
 pgfcore.code.tex
pgfbaseshapes.sty    2006/10/16 v1.10 (rcs-revision 1.16)
pgfbaseshapes.code.tex
pgfbaseplot.sty    2006/10/16 v1.10 (rcs-revision 1.5)
pgfbaseplot.code.tex
pgfbaseimage.sty    2006/10/16 v1.10 (rcs-revision 1.5)
pgfbaseimage.code.tex
pgfbaselayers.sty    2006/10/16 v1.10 (rcs-revision 1.5)
pgfbaselayers.code.tex
pgfbasesnakes.sty    2006/10/16 v1.10 (rcs-revision 1.10)
pgfbasesnakes.code.tex
pgfbasepatterns.sty    2006/10/16 v1.10 (rcs-revision 1.9)
pgfbasepatterns.code.tex
pgfcomp-version-0-65.sty    2006/10/11 v1.10 (rcs-revision 1.4)
    calc.sty    2005/08/06 v4.2 Infix arithmetic (KKT,FJ)
  pgffor.sty    2006/10/16 v1.10 (rcs-revision 1.5)
  pgffor.code.tex
    tikz.code.tex
 amsmath.sty    2000/07/18 v2.13 AMS math features
 amstext.sty    2000/06/29 v2.01
  amsgen.sty    1999/11/30 v2.0
  amsbsy.sty    1999/11/29 v1.2d
  amsopn.sty    1999/12/14 v2.01 operator names
colortbl.sty    2001/02/13 v0.1j Color table columns (DPC)
   array.sty    2005/08/23 v2.4b Tabular extension package (FMi)
listings.sty    2004/10/17 1.3b (Carsten Heinz)
lstpatch.sty    2004/10/17 1.3b (Carsten Heinz)
 lstmisc.sty    2004/09/07 1.3 (Carsten Heinz)
listings.cfg    2004/09/05 1.3 listings configuration
lstlang1.sty    2004/09/05 1.3 listings language file
lstlang1.sty    2004/09/05 1.3 listings language file
 upquote.sty    2003/08/11 v1.1 Covington's upright-quote modification to verba
tim and verb
textcomp.sty    2005/09/27 v1.99g Standard LaTeX package
  ts1enc.def    2001/06/05 v3.0e (jk/car/fm) Standard LaTeX file
  ts1cmr.fd    1999/05/25 v2.5h Standard LaTeX font definitions
   t1phv.fd    2001/06/04 scalable font definitions for T1/phv.
supp-pdf.tex
ragged2e.sty    2003/03/25 v2.04 ragged2e Package (MS)
everysel.sty    1999/06/08 v1.03 EverySelectfont Package (MS)
 nameref.sty    2006/12/27 v2.28 Cross-referencing by name of section
refcount.sty    2006/02/20 v3.0 Data extraction from references (HO)
hic-manual_base.out
hic-manual_base.out
 ot1pplx.fd    2004/09/06 font definitions for OT1/pplx.
 omlzplm.fd    2002/09/08 Fontinst v1.914 font definitions for OML/zplm.
 omszplm.fd    2002/09/08 Fontinst v1.914 font definitions for OMS/zplm.
 omxzplm.fd    2002/09/08 Fontinst v1.914 font definitions for OMX/zplm.
 ot1zplm.fd    2002/09/08 Fontinst v1.914 font definitions for OT1/zplm.
figure/NYU_Langone.jpg
child/Introduction/install-setup-run.tex
  t1cmtt.fd    1999/05/25 v2.5h Standard LaTeX font definitions
figure/sample_sheet_screenshot.png
child/Introduction/dependencies.tex
child/default-pipeline-components.tex
  ts1phv.fd    2001/06/04 scalable font definitions for TS1/phv.
child/code-structure.tex
child/auto_report.tex
child/custom_pipeline_step.tex
child/HiC/index.tex
child/HiC/align.tex
child/HiC/filter.tex
child/HiC/filter-stats.tex
figure/filter-stats_counts.pdf
figure/filter-stats_percent.pdf
child/HiC/tracks.tex
child/HiC/matrix-filtered.tex
child/HiC/matrix-prep.tex
```

```
child/HiC/matrix-ic.tex
child/HiC/matrix-hicnorm.tex
child/HiC/matrix-stats.tex
figure/matrix-stats_stats.pdf
child/HiC/compare-matrices.tex
child/HiC/compare-matrices-stats.tex
figure/compare-matrices-stats_correlograms.pdf
figure/compare-matrices-stats_pearson_correlograms.pdf
child/HiC/boundary-scores.tex
child/HiC/boundary-scores-pca.tex
figure/boundary-scores-pca_pca_DI_k_001.pdf
child/HiC/domains.tex
child/HiC/compare-boundaries.tex
child/HiC/compare-boundaries-stats.tex
figure/compare-boundaries-stats_raw_comparisons.pdf
figure/compare-boundaries-stats_correlograms.pdf
child/HiC/hicplotter.tex
figure/hicplotter_chr8-125000000-133000000.pdf
child/HiC/interactions.tex
figure/interactions_plots.pdf
child/HiC/annotations.tex
child/HiC/annotations-stats.tex
figure/annotations-stats_enrichment.pdf
child/Appendix/appendix.tex
 ts1cmtt.fd    1999/05/25 v2.5h Standard LaTeX font definitions
```
