# Supplementary figures and images for: HiC-bench: comprehensive and reproducible Hi-C data analysis designed for parameter exploration and benchmarking
Source: BMC Genomics. 2017 Jan 5;18:22. doi: 10.1186/s12864-016-3387-6 (PMC5217551; doi:10.1186/s12864-016-3387-6)

# Supplementary Figure 1

A

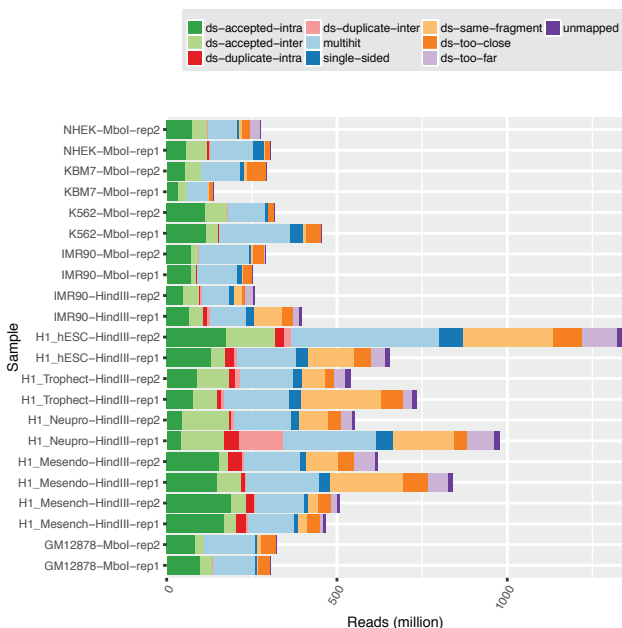

B

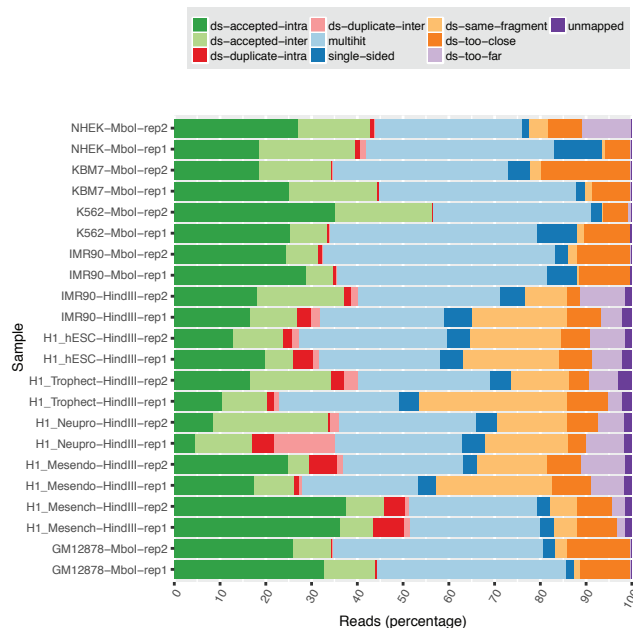

Supplement: Additional file 4: Figure S1. — Hi-C reads filtering statistics. Number (A) and percentage (B) of the various read categories identified during filtering for all datasets used in the study. Mappable reads were over 95% in all samples. Duplicate (ds-duplicate-intra and ds-duplicate-inter; red and pink respectively), non-uniquely mappable (multihit; light blue), single-end mappable (single-sided; dark blue) and unmapped reads (unmapped; dark purple) were discarded. Self-ligation products (ds-same-fragment; orange) and reads mapping too far (ds-too-far; light purple) from restriction sites or too close to one another (ds-too-close; orange) were also discarded. Only double-sided uniquely mappable cis (ds-accepted-intra; dark green) and trans (ds-accepted-inter; light green) read pairs were used for downstream analysis. The x axis represents either the raw read number (A) or the percentage of reads (B) falling within each of the categories described in the legend. The y axis represents the samples. (PDF 1380 kb) [file 12864_2016_3387_MOESM4_ESM.pdf]

Supplementary Figure 2

Filtered

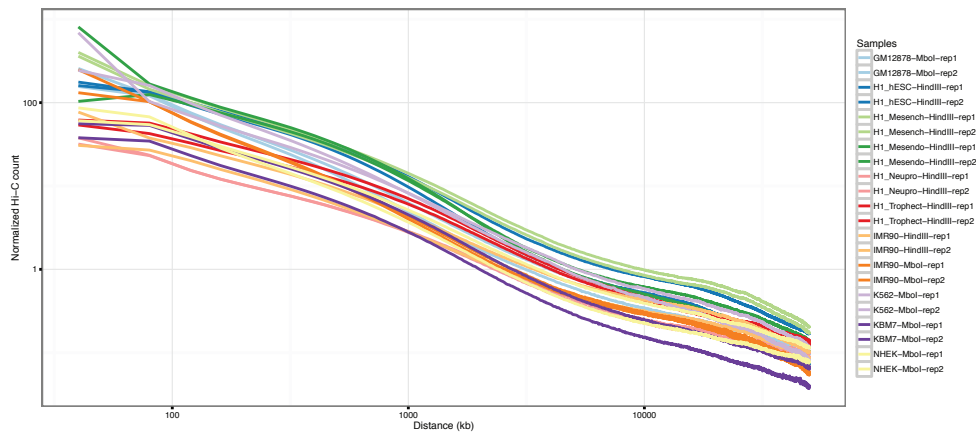

IC

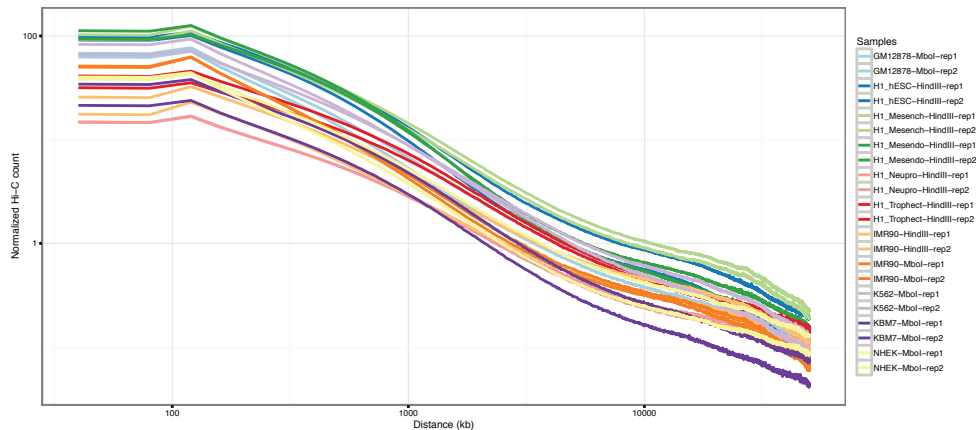

Supplement: Additional file 5: Figure S2. — Matrix statistics. Normalized Hi-C counts are presented as a function of the distance between the interacting partners for all samples and correction methods. The Hi-C samples analyzed were GM12878 (light blue), hESCs (H1) (blue), mesenchymal cells (light green), mesendoderm (dark green), neural progenitors (pink), trophectoderm (red), IMR90 (light and dark orange), K562 (light purple), KBM7 (dark purple) and NHEK (yellow). The matrices were either unprocessed (filtered) (top) or corrected using IC (bottom). The y axis represents the normalized count of Hi-C interactions and the x axis the distance between the interacting partners in kilobases. (PDF 2050 kb) [file 12864_2016_3387_MOESM5_ESM.pdf]

Filtered

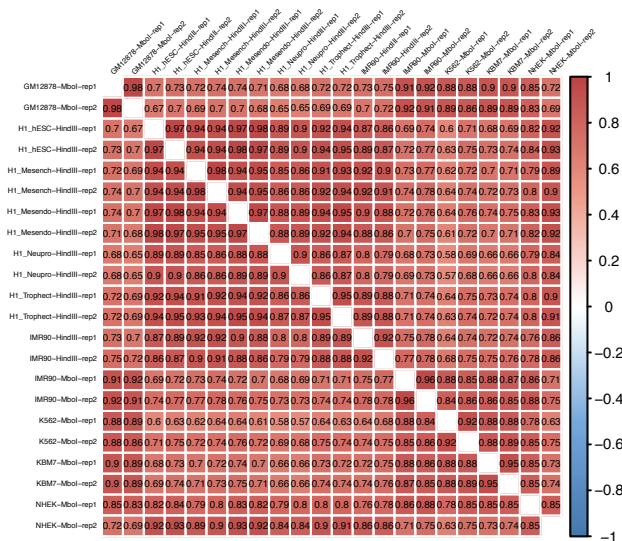

IC

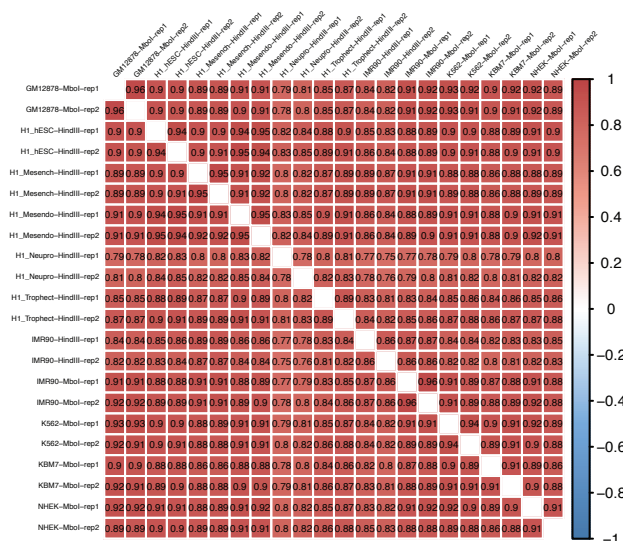

Supplement: Additional file 6: Figure S3. — Pairwise Pearson correlation of Hi-C matrices. Correlograms summarizing all pairwise Pearson correlations for all Hi-C samples used in this study: raw (filtered) matrices (left panel) and matrices after iterative correction (right panel). Dark red indicates strong positive correlation and dark blue strong negative. The resolution of the matrices is 40 kb. (PDF 1405 kb) [file 12864_2016_3387_MOESM6_ESM.pdf]

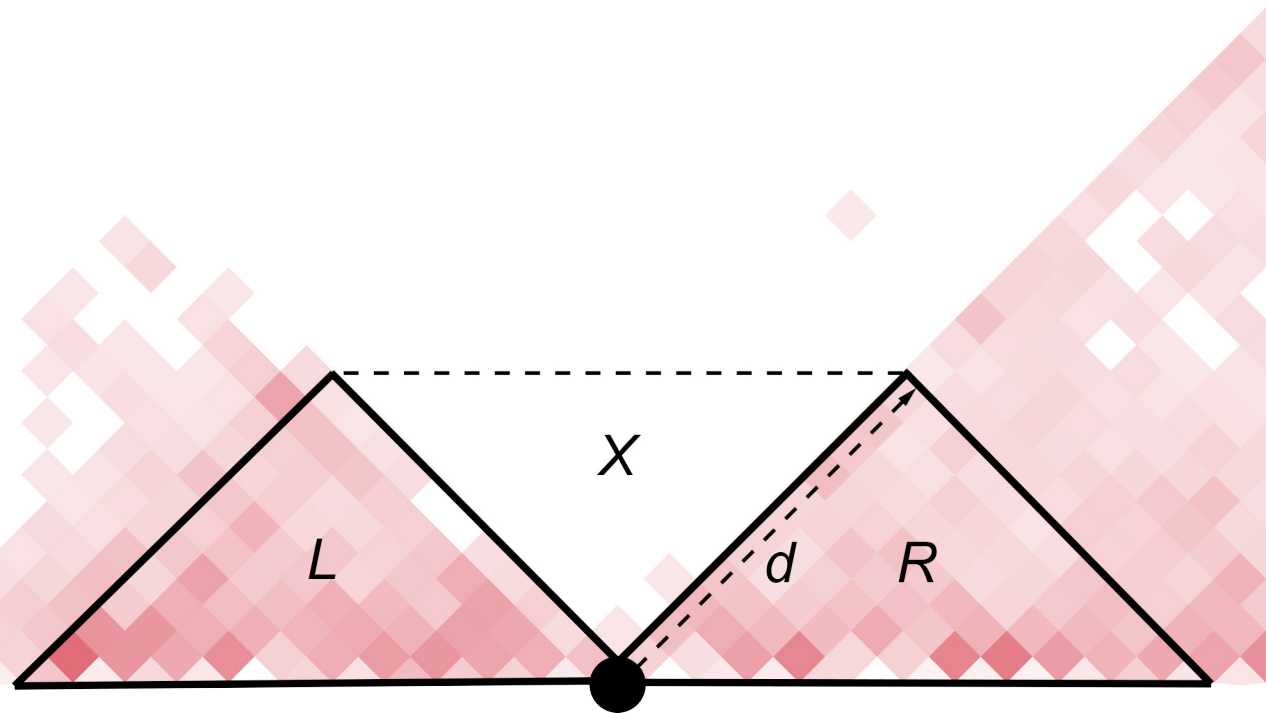

Supplement: Additional file 7: Figure S4. — Boundary score calculation. Two adjacent topological domains (red triangles) are depicted. The left domain (L) is separated from the right domain (R) by a boundary (black circle). The areas of more-frequent intra-domain interactions are in red. The area of less-frequent cross-domain (or inter-domain) interactions is X. We also introduce parameter d which is the maximum distance from the diagonal to be considered for the calculation of boundary scores (default value: d = 2 Mb). (PDF 1546 kb) [file 12864_2016_3387_MOESM7_ESM.pdf]

Supplementary Figure 5

Filtered

Ratio

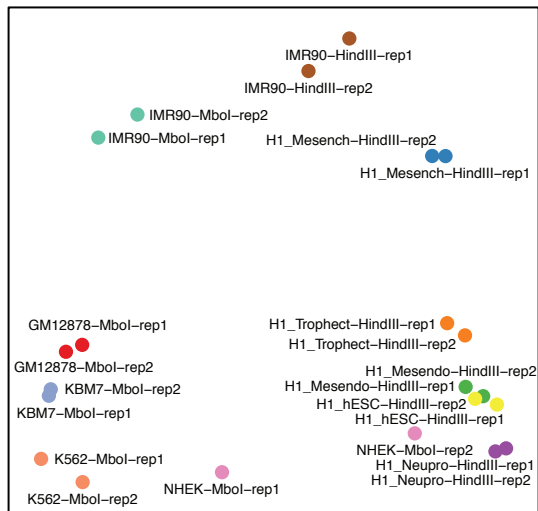

IC

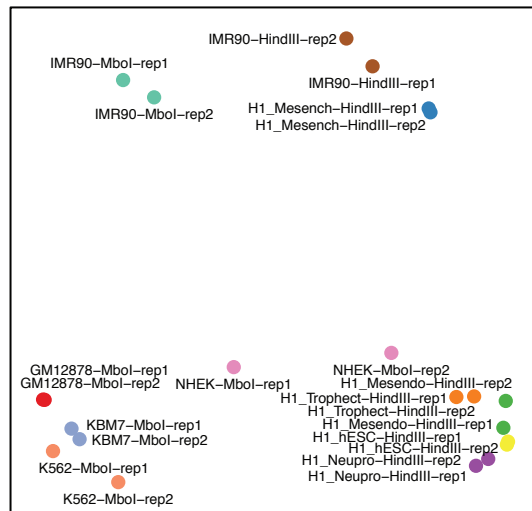

PC2  
PC1

Supplement: Additional file 8: Figure S5. — Principal component analysis of boundary scores. Boundary scores were calculated using ratio score, for all samples either before (filtered) (left panel) or after iterative correction (IC) (right panel). (PDF 882 kb) [file 12864_2016_3387_MOESM8_ESM.pdf]

Filtered

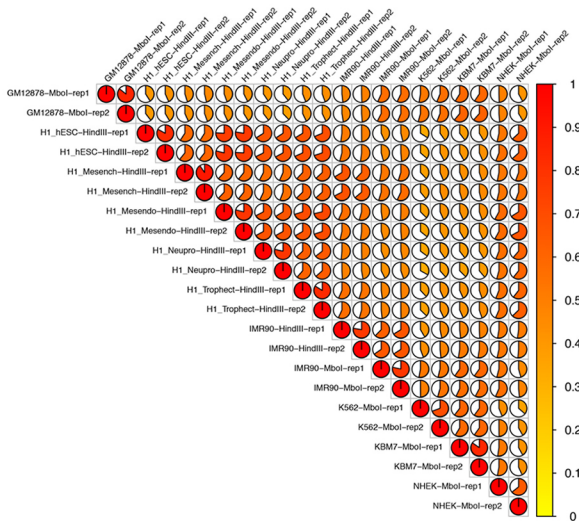

IC

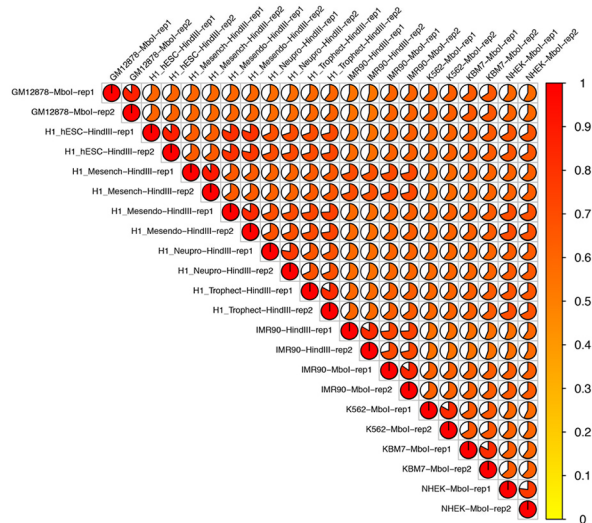

Supplement: Additional file 9: Figure S6. — Pairwise overlaps of TAD boundaries. The pairwise overlaps of TAD boundaries are shown for all samples of this study, after calling boundaries using hicratio (all reads, d = 0500). Before TAD calling, the Hi-C matrices were either unprocessed (filtered) or corrected using iterative correction (IC) (resolution = 40 kb). (PDF 3847 kb) [file 12864_2016_3387_MOESM9_ESM.pdf]

IC

Filtered

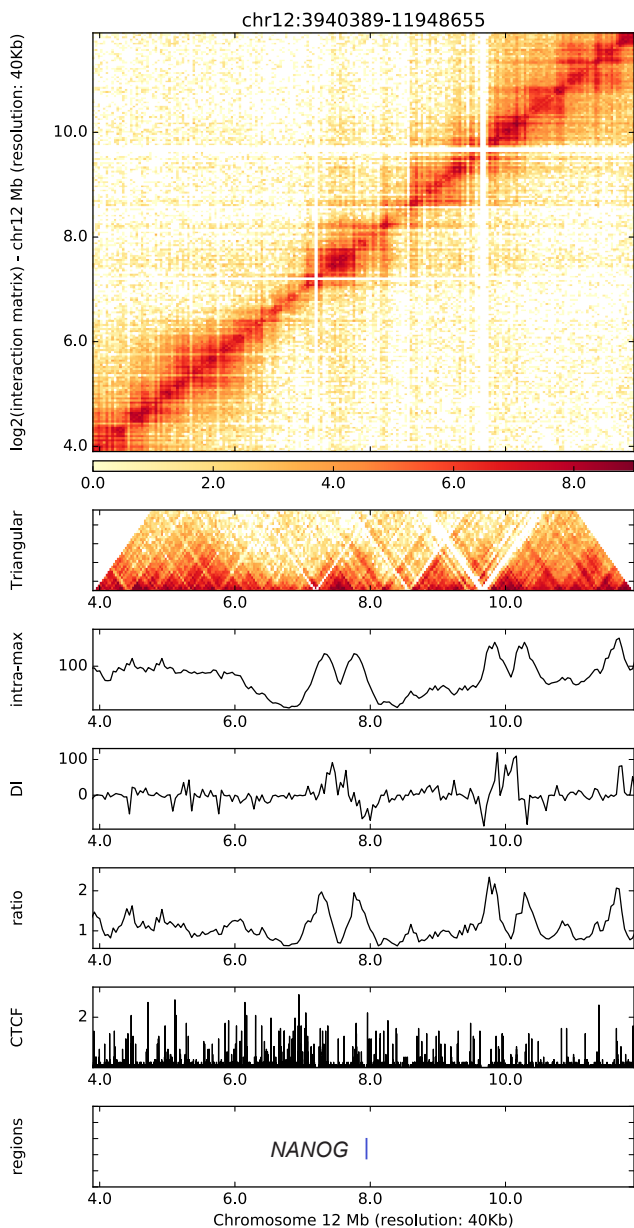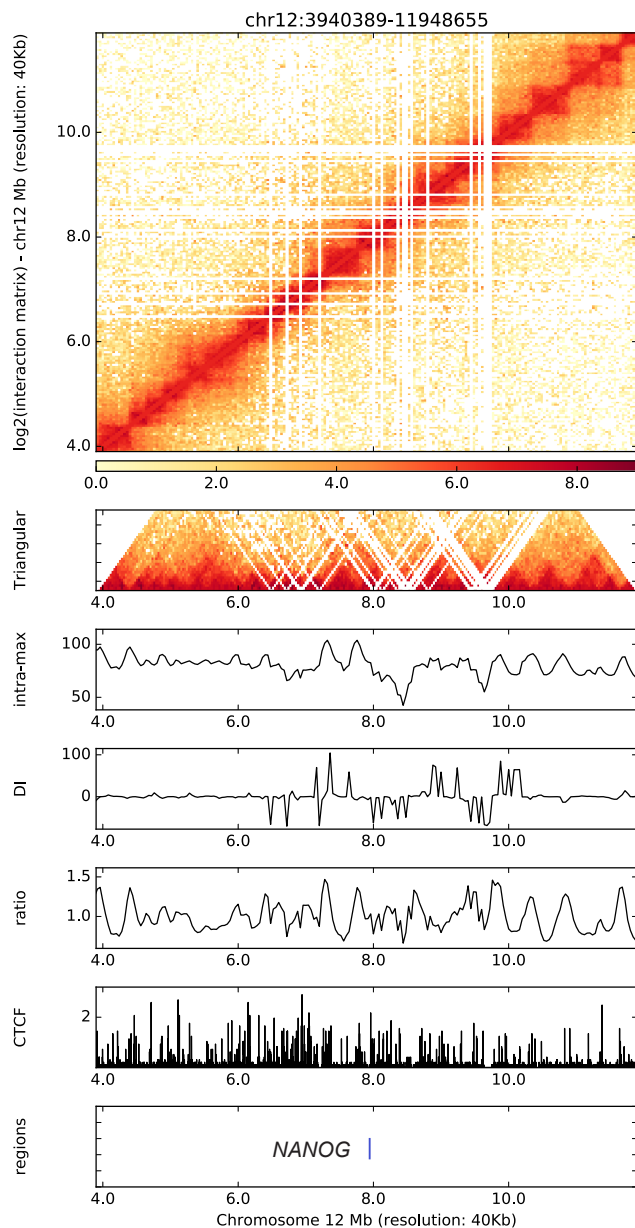

Supplement: Additional file 10: Figure S7. — Visualization of TADs and certain areas of interest. HiC-bench integrates HiCPlotter [23] and it offers the ability to easily prepare publication-quality figures. We present the area surrounding NANOG, a gene of particular importance for the maintenance of pluripotency. The Hi-C matrix corresponding to the chr12:3940389–11948655 genomic region is presented for H1 cells, before and after matrix correction. The matrix is also rotated 45° to facilitate TAD visualization. Various boundary scores (intra-max, DI, ratio) are shown as individual tracks along with CTCF binding. The location of NANOG is presented as a blue line. (PDF 1307 kb) [file 12864_2016_3387_MOESM10_ESM.pdf]

## IMR90 sample

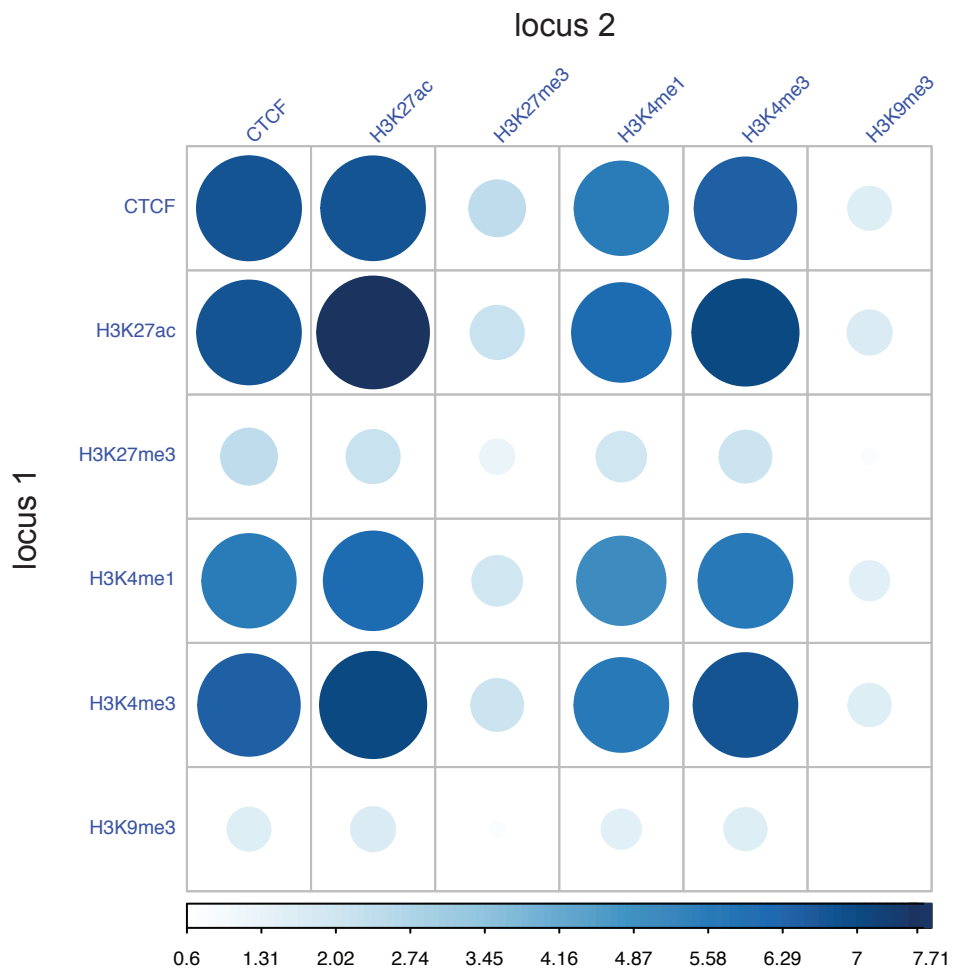

## H1 sample

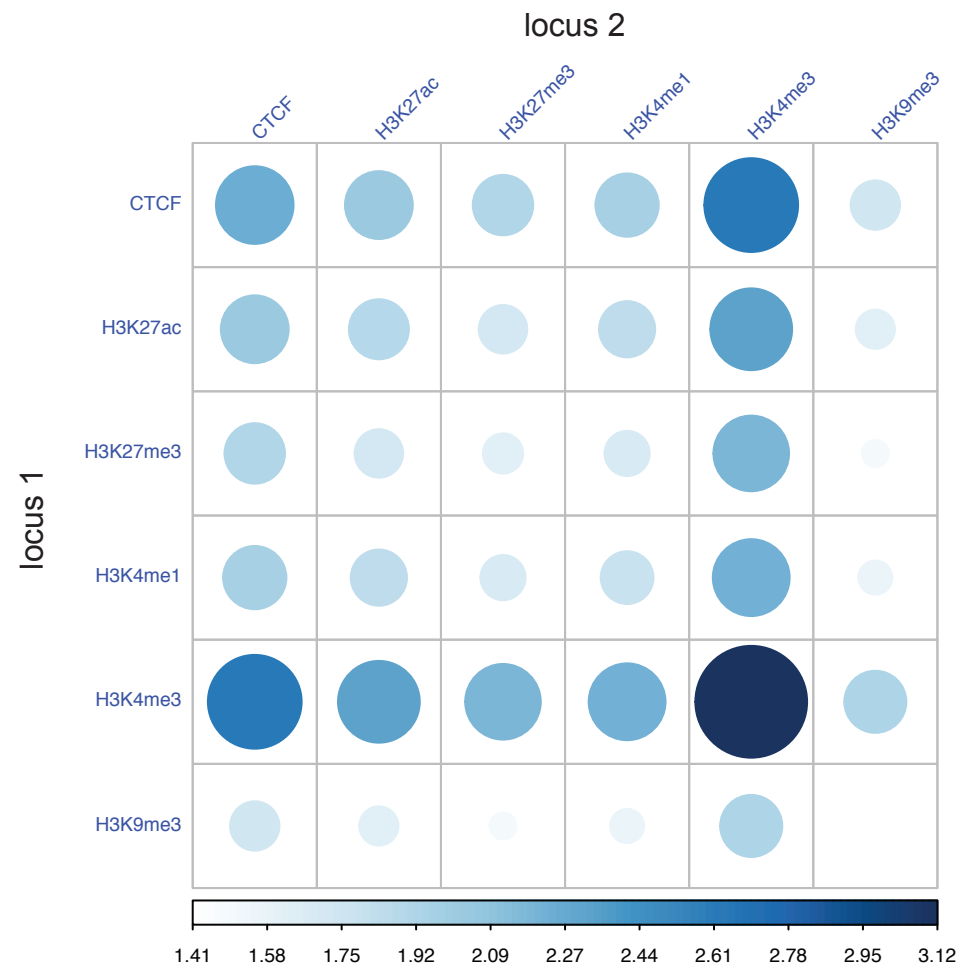

Supplement: Additional file 11: Figure S8. — Enrichment of chromatin interactions in human fibroblasts (IMR90) and embryonic stem cells (H1). The enrichment of certain chromatin marks and CTCF in the top 50,000 chromatin interactions in the IMR90 and H1 samples is shown. Deep blue and larger circle size indicate higher enrichment. (PDF 921 kb) [file 12864_2016_3387_MOESM11_ESM.pdf]
